# Geometrical Influence on Particle Transport in Cross-Flow Ultrafiltration: Cylindrical and Flat Sheet Membranes

**DOI:** 10.3390/membranes11120960

**Published:** 2021-12-06

**Authors:** Gun Woo Park, Gerhard Nägele

**Affiliations:** Institute of Biological Information Processing (IBI-4), Forschungszentrum Juelich GmbH, 52425 Jülich, Germany; g.naegele@fz-juelich.de

**Keywords:** ultrafiltration, cross-flow filtration, concentration-polarization, membrane geometry, hollow fiber, tubular membrane, flat sheet membrane, plate-and-frame

## Abstract

Cross-flow membrane ultrafiltration (UF) is used for the enrichment and purification of small colloidal particles and proteins. We explore the influence of different membrane geometries on the particle transport in, and the efficiency of, inside-out cross-flow UF. For this purpose, we generalize the accurate and numerically efficient modified boundary layer approximation (mBLA) method, developed in recent work by us for a hollow cylindrical membrane, to parallel flat sheet geometries with one or two solvent-permeable membrane sheets. Considering a reference dispersion of Brownian hard spheres where accurate expressions for its transport properties are available, the generalized mBLA method is used to analyze how particle transport and global UF process indicators are affected by varying operating parameters and the membrane geometry. We show that global process indicators including the mean permeate flux, the solvent recovery indicator, and the concentration factor are strongly dependent on the membrane geometry. A key finding is that irrespective of the many input parameters characterizing an UF experiment and its membrane geometry, the process indicators are determined by three independent dimensionless variables only. This finding can be very useful in the design, optimization, and scale-up of UF processes.

## 1. Introduction

Membrane ultrafiltration (UF) is used in daily life, e.g., in water purification, blood treatment by (artificial) kidneys, and protein enrichment [1,2,3]. It is the pressure-driven membrane filtration of smaller, sub-micron sized particles dispersed in a low-molecular solvent (mostly water) under conditions of larger trans-membrane pressure (TMP) values. Owing to strong Brownian motion of the particles, the dispersion remains practically in thermodynamic equilibrium during UF, and there is a significant osmotic pressure buildup along the membrane surface. This distinguishes UF from microfiltration of larger, typically micron-sized particles where Brownian motion and (equilibrium) osmotic pressure effects are negligible. Instead, non-equilibrium micro-hydrodynamic effects including shear-induced collective diffusion are of importance in microfiltration.

UF is often performed using a cross-flow setup, where a feed dispersion is steadily pumped through a parallel bundle of membrane modules having inlet and outlet ports. The particles-enriched dispersion is collected at the outlet ports. Driven by the TMP, there is a non-homogeneous, particles-enriched diffuse layer formed near the surface of a membrane retaining the particles. This concentration-polarization (CP) layer becomes, in general, more pronounced with increasing distance from the inlet port. The CP layer is determined by the balance of gradient (collective) diffusion of particles away and by the flow advection towards the membrane surface. The enlarged dispersion viscosity inside the CP layer further enhances polarization. The osmotic pressure buildup caused by the CP layer counteracts the applied TMP, which in turn lowers the filtration efficiency.

In addition to CP layer formation, there are unwarranted membrane fouling effects, which lower the filtration efficiency. Fouling is caused by specific physico-chemical interactions between particles and membranes, implying, e.g., stagnant cake layer formation, particle adsorption at the membrane wall, or clogging of the membrane pores [3,4,5,6,7]. The present work focuses on the influence of different membrane geometries on particle transport and generic CP layer formation in UF. We are not dealing here with specific fouling effects. The considered UF operating conditions are such that reversible cake layer formation due to crystallization or jamming is avoided.

**Figure 1:**
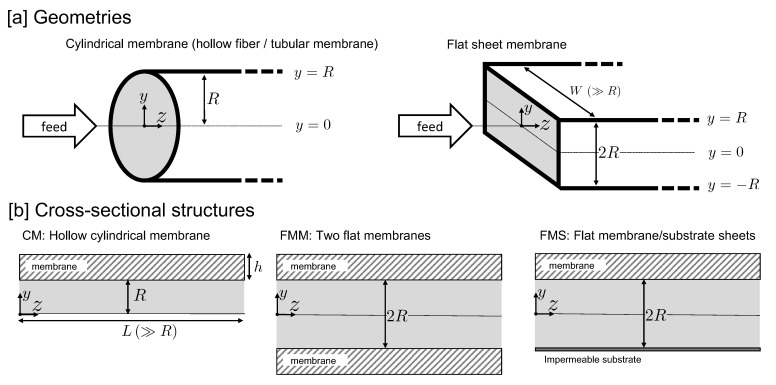
Considered membrane geometries: (**a**) cylindrical membrane of inner radius *R* (left), and flat sheets membranes of height 2R and width W≫R (right). (**b**) Cross-sectional structure of a cylindrical membrane (left, CM), a membrane consisting of an upper and lower flat sheet part at vertical distance 2R (middle, FMM), and a flat membrane–flat substrate combination where the bottom substrate sheet is impermeable to particles and solvent (right, FMS). The membrane thickness is *h*, and *L* with L≫R is the axial length of the membrane.

Among the different membrane geometries encountered in UF setups, there are two common ones depicted in Figure 1, which are discussed in the present work. The first one is the standard cylindrical shape of a hollow fiber or tubular membrane having circular cross-section, characterized by an inner membrane radius *R* and axial length *L* with R≪L. This is referred to in what follows as a cylindrical membrane (CM). The second one is a flat sheets geometry with a rectangular cross-section of height 2R and width *W*, where R≪W and R≪L. Here, *L* denotes again the axial length of the membrane. Regarding the flat sheets geometry, we differentiate between two types of membranes. The first type consists of two parallel flat membrane sheets of area W·L each, referred to as the FMM membrane. The second one has a top membrane sheet and a non-permeable bottom sheet referred to as the substrate sheet (c.f. Figure 1). We refer to this asymmetric flat sheets membrane system as an FMS membrane system.

The CM membrane is distinguished from the FMM membrane by a larger cross-sectional circumference-to-area ratio of 2/R, while this ratio was equal to 1/R in the FMM case. For otherwise equal membrane properties, i.e., equal hydraulic permeability $L_{p}$, *R*, *L*, and for equal TMP and mean inlet velocity, this difference in the ratio implies a larger solvent recovery for the CM membrane. In spite of being less fouling-resistant than CM membranes, FMM and FMS membrane systems are often used as plate-and-frame setups in industrial applications, owing to their compact design and being less expensive. Moreover, flat sheet membranes can be operated at larger TMP, since they are commonly supported mechanically by plates or rigid porous spacers on the permeate side [8,9,10]. The FMS module with a transparent substrate sheet is also useful for monitoring flow behavior and fouling [11,12,13,14].

In this work, we analyze differences and similarities in particle transport and flow efficiency of CM, FMM, and FMS membrane systems under UF conditions. For simplicity, we assume that the membranes are ideally particle retentive and that the particles are mono-disperse colloidal hard spheres. For fluid-like hard-sphere dispersions, accurate analytic expressions are available for the equilibrium gradient-diffusion coefficient and viscosity, and for the osmotic pressure. To calculate steady-state flow properties and particle concentration profiles, we use a versatile, semi-analytic modified boundary-layer approximation (mBLA) method, developed in earlier work by us for CM membranes [15], and we generalize it to FMM and FMS geometries. It was shown in [15] that this method provides concentration and flow profiles in excellent agreement with the according results obtained from elaborate finite-element calculations. In the mBLA method, the flow outside the CP boundary layer is determined basically by the, in general, hyperbolic axial pressure profile caused by the permeate flux through the membrane, as described in [16,17,18]. The inner flow solution is obtained in a similar way as in classical film theory [4,5,19,20], however with the concentration-dependence of the dispersion properties accounted for. The mBLA method is computationally fast, and different from computational fluid dynamics methods, it offers analytic insight into the functional behavior of concentration and flow properties. In this context, we note that approximate solutions for pure solvent flow in a FMM channel are discussed in [21,22] and for dispersion flow in [23,24,25].

For varied operating conditions, we investigate how the CM, FMM, and FMS geometries affect the concentration, dispersion flux profiles, and global process indicators, including the mean permeate flux and the solvent recovery indicator. The mBLA results recover, in particular, the experimentally observed dependence of the mean permeate flux on the logarithm of the feed concentrations [9]. A major finding of this study is the dependence of the solvent-recovery indicator on a minimal set of only three input variables, which applies to all considered membrane geometries.

The article is organized as follows. In Section 2, we summarize analytic expressions for the suspension transport properties and osmotic pressure, used as input to the generalized mBLA method. The employed cross-flow UF model and its underlying transport equations are discussed in Section 3. Furthermore, the respective boundary conditions for the CM, FMM, and FMS geometries are explained. Section 4 gives the essentials of the mBLA method generalized to the FMM and FMS geometries, and it includes a discussion of its pros and cons. Our results are presented in Section 5. Results for the CP layer profile and particle transport for fixed TMP are discussed in Section 5.1. Global-process indicators are analyzed in Section 5.2 for varying TMP. The minimal set of the three variables fully characterizing the global process indicators is presented in Section 5.3 and discussed in conjunction with Appendix A. Our conclusions are contained in Section 6.

## 2. Dispersion of Brownian Hard Spheres

The scope of this study is to analyze the influence of three different membrane geometries on generic CP layer effects and the UF efficiency. Since we were not concerned here with effects on UF arising from specific particle interactions, as a reference dispersion, we employ the model of mono-disperse Brownian hard spheres whose thermodynamic and transport properties are characterized by two parameters only, namely, the volume fraction $\phi =\left(4\pi /3\right)na^{3}$ and the hard-core radius *a*. Here, *n* is the number density of spheres.

**Figure 2:**
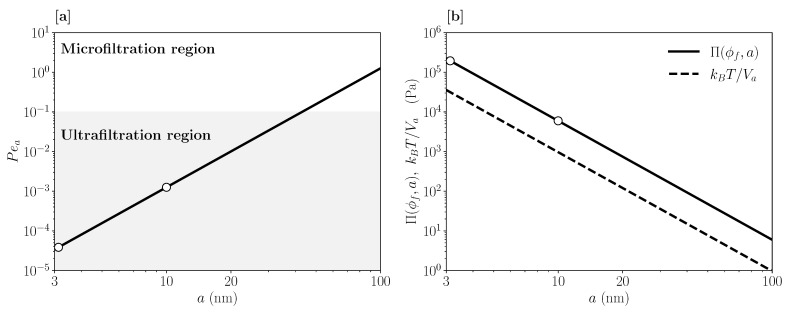
(**a**) Single-particle shear-Pèclet number, $Pe_{a}\propto a^{3}$, as a function of particle radius *a* and for a shear rate ${\dot{\gamma }}^{*}=270/s$ typical of UF. (**b**) Carnahan–Starling-based osmotic pressure at freezing, $\Pi (\phi_{f},a)$, and thermal pressure, $k_{B}T/V_{a}$, as functions of particle radius *a*. Open circles indicate the radii a=3.13 nm and a=10 nm used in this work.

The flow conditions considered in this work are such that the single-particle Pèclet number, $Pe_{a}$, obeys [26,27]

(1) $$Pe_{a}\equiv \frac{{\dot{\gamma }}^{*}a^{2}}{D_{0}}\ll 1\;,$$

where ${\dot{\gamma }}^{*}$ is a characteristic shear rate, taken as the shear rate of the velocity field at the inner membrane wall right at the inlet. Furthermore, $D_{0}=k_{B}T/\left(6\pi \eta_{s}a\right)$ is the single-particle Stokes–Einstein diffusion coefficient for stick hydrodynamic boundary conditions [26], and $\eta_{s}$ is the viscosity of the Newtonian solvent. For small $Pe_{a}$, the dispersion is only slightly perturbed away from equilibrium (see Figure 2a), with the Brownian forces acting on the particles dominating the viscous ones. This characterizes the UF region, marked by the shaded area in Figure 2a. To model UF, one is allowed to use the equilibrium forms of the transport coefficients and osmotic pressure for a quiescent dispersion, for which accurate analytic expressions are available.

The equilibrium osmotic pressure, Π, of Brownian hard spheres can be described by the Carnahan–Starling expression [28,29]

(2) $$\Pi \left(\phi ,a\right)=\frac{k_{B}T}{V_{a}}\phi \frac{1+\phi +\phi^{2}-\phi^{3}}{{\left(1-\phi \right)}^{3}}\;,$$

where $V_{a}=\left(4\pi /3\right)a^{3}$ is the particle volume, and ϕ is the particle volume fraction. This expression holds to high accuracy up to the freezing transition volume fraction $\phi_{f}\approx 0.494$. In Figure 2b, the maximal fluid-state osmotic pressure reached at freezing, $\Pi (\phi_{f},a)$, is plotted as a function of the particle radius *a* in conjunction with the thermal pressure $k_{B}T/V_{a}$, where $k_{B}$ is the Boltzmann constant, and *T* is the dispersion temperature. Notice that the $1/a^{3}$ dependence of both quantities implies that the osmotic pressure is strongly reduced with increasing particle size.

**Figure 3:**
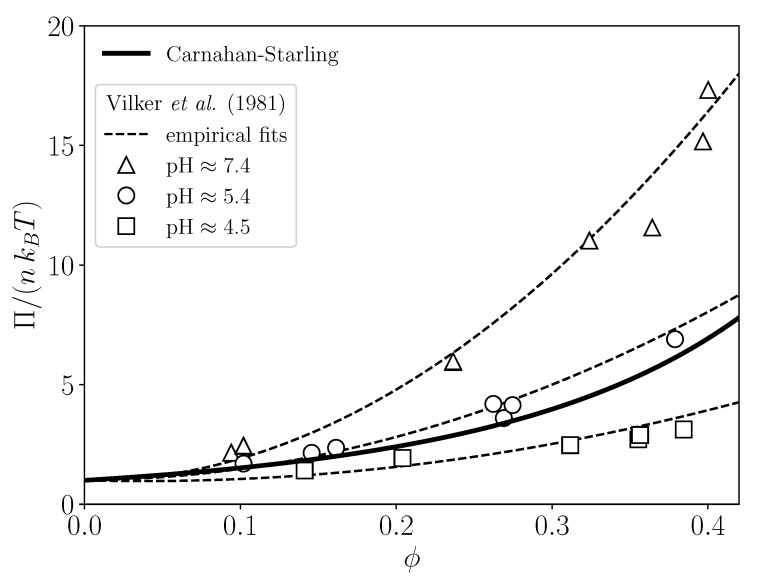
Reduced osmotic pressure, $\Pi /(nk_{B}T)$, as a function of particle volume fraction ϕ. The solid line is the Carnahan–Starling prediction for hard spheres, compared to experimental data (open symbols) for bovine serum albumin solutions at different pH values as indicated. Experimental data are reproduced from [30]. Dashed lines are empirical fits to the data.

To illustrate that the Carnahan–Starling expression for neutral hard spheres is a decent description also for aqueous solutions of globular proteins near the isoelectric point; in Figure 3, we depict osmotic pressure data by Vilker et al. [30] as a function of ϕ, for bovine serum albumin solutions at three different pH values. As noted in [30], the isoelectric pH value is about 4.72 in a 0.15 M saline solution. We determined the volume fractions ϕ in Figure 3 from the protein concentration values and the equivalent spherical radius value a=3.13 nm given in [30]. The pressure data at pH≈4.5 are located below the Carnahan–Starling curve, suggesting that there is an attractive interaction contribution.

The gradient diffusion coefficient, *D*, i.e., the long-time collective diffusion coefficient, is only slightly smaller than the associated short-time collective diffusion coefficient. Hence, we can approximate *D* to good accuracy by its short-time form equal to the right-hand-side of [27,29,31]

(3) $$D\left(\phi \right)\approx D_{0}\frac{K^{sed}\left(\phi \right)}{S(\phi )}\;,$$

where $K^{sed}$ is the short-time sedimentation coefficient, $D_{0}$ is the single-particle diffusion coefficient, and S(ϕ) is the osmotic compressibility factor. For the sedimentation coefficient of Brownian hard spheres, we use the analytic expression [31]

(4) $$K^{sed}\left(\phi \right)=1-6.5464\;\phi \;\left[1-3.348\phi +7.426\phi^{2}-10.034\phi^{3}+5.882\phi^{4}\right].$$

**Figure 4:**
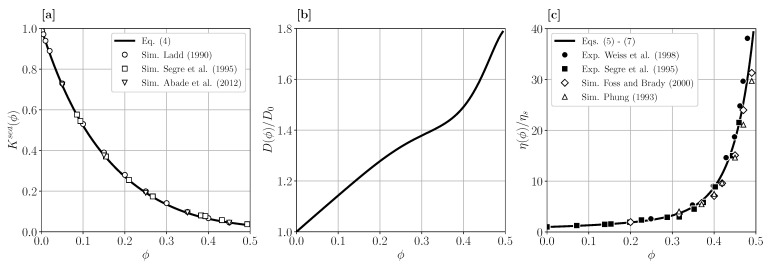
(**a**) Concentration dependence of the short-time sedimentation coefficient $K^{sed}\left(\phi \right)$. Solid line is the prediction by Equation (4), and open symbols are dynamic simulation data by three groups as indicated [32,33,34]. (**b**) Short-time collective diffusion coefficient, D(ϕ), according to Equation (3) (solid line), given in units of $D_{0}$. (**c**) Dispersion shear viscosity, η(ϕ), in units of the solvent viscosity $\eta_{s}$. Solid line is the prediction by Equations (5)–(7). Open symbols are dynamic simulation and filled symbols experimental viscosity data reproduced from Refs. [35,36] and Refs. [33,37], respectively.

As shown in Figure 4a, this expression is in excellent agreement with according dynamic simulation data where many-particles hydrodynamic interactions are accounted for. For the inverse of the osmotic compressibility factor, S(ϕ), the accurate Carnahan–Starling-type expression $1/S\left(\phi \right)=\left[{\left(1+2\phi \right)}^{2}+\phi^{3}\left(\phi -4\right)\right]/{\left(1-\phi \right)}^{4}$ is used [29]. Figure 4b displays the according (short-time) gradient diffusion coefficient, which grows monotonically with increasing particle volume fraction. The gradient diffusion coefficient, D(ϕ), should not be confused with the self-diffusion coefficient, $D_{S}\left(\phi \right)$. In fact, the latter coefficient decreases with increasing concentration below its infinite dilution value, $D_{0}$, both for attractive and repulsive interactions.

The dispersion shear viscosity, η, with [27]

(5) $$\eta \left(\phi \right)=\eta_{\infty }\left(\phi \right)+\Delta \eta \left(\phi \right),$$

is the sum of a high-frequency (short-time) viscosity part, $\eta_{\infty }$, and a shear relaxation viscosity part, Δη. The latter viscosity contribution accounts for the non-instantaneous, visco-elastic response of the dispersion microstructure to the locally generated shear flow. For Brownian hard spheres at low $Pe_{a}$, the two viscosity contributions are approximated, to good accuracy up to the freezing volume fraction, by [31]

(6) $$\begin{array}{cc} \eta_{\infty }\left(\phi \right)/\eta_{s} & =1+\frac{5}{2}\phi \frac{1+\hat{S}\left(\phi \right)}{1-\phi (1+\hat{S}\left(\phi \right))} \end{array}$$

(7) $$\begin{array}{cc} \Delta \eta \left(\phi \right)/\eta_{s} & =\frac{D_{0}}{D_{s}\left(\phi \right)}\frac{12\phi^{2}\left(1-7.085\phi +20.182\phi^{2}\right)}{5(1-\phi /0.64)}\;, \end{array}$$

with the Saito-type function $\hat{S}\left(\phi \right)=\phi \left(1+0.95\;\;\phi -2.15\;\;\phi^{2}\right)$. Here, $D_{s}\left(\phi \right)/D_{0}=1-1.8315\phi \left(1+0.12\phi -0.70\phi^{2}\right)$ is a good analytic approximation for the short-time self-diffusion coefficient of Brownian hard spheres. Up to a factor of six, $D_{s}\left(\phi \right)$ quantifies the initial slope of the mean squared displacement of hydrodynamically interacting particles. In Figure 4c, the concentration-dependent viscosity expression according to Equations (5)–(7) is depicted with simulation and experimental data. There is good agreement in the fluid-phase regime of the dispersion.

For cross-flow operating conditions compatible with the considered channel geometries, there is dispersion flow without swirling. The dispersion-averaged velocity field, V, is accordingly of the form

(8) $$V\left(y,z\right)=v\left(y,z\right)\;\;\hat{y}+u\left(y,z\right)\;\;\hat{z}\;,$$

where $\hat{z}$ is the unit vector in axial direction. For the CM channel, $\hat{y}$ is the radial unit vector pointing from the inner axis to the cylindrical membrane wall, whereas for the FMM and FMS channels, $\hat{y}$ is the unit vector in the transversal direction, perpendicular to the parallel sheets (c.f. Figure 1). Furthermore, v(y,z) is the transversal, and u(y,z) is the axial velocity component.

The membrane is assumed to be fully particle retentive to the Brownian spheres. Tangential stick fluid boundary conditions are used at the inner membrane wall(s), since, in UF, the axial (i.e., *z* direction) solvent flow velocity inside a membrane is distinctly smaller than the axial dispersion flow velocity inside the lumen.

## 3. Modeling Concentration-Polarization in Ultrafiltration

Consider a dispersion of mono-disperse Brownian spheres steadily pumped through a CM, FMM, or FMS conduit as illustrated in Figure 1. We describe the dispersion flow on a coarse-grained level where the particle size and the porous structure of the membrane remain unresolved. The mass and momentum transport in UF are then described by macroscopic continuum mechanics equations governing the spatio-temporal evolution of the dispersion-averaged volume concentration field ϕ(r,t) of particles at position r and time *t*, and the dispersion-averaged velocity field V(r,t).

For conditions met in UF where the single-particle Reynolds number is small compared to one, the dispersion-averaged incompressible laminar flow is described by the quasi-stationary effective Stokes equation [26,27],

(9) $$\begin{array}{ccc} \nabla P & = & \eta \left(\phi \right)\Delta V+\frac{d\eta }{d\phi }\nabla \phi \cdot \left[\nabla V+{\left(\nabla V\right)}^{T}\right] \end{array}$$

(10) $$\begin{array}{ccc} \nabla \cdot V & = & 0\;, \end{array}$$

in conjunction with the incompressibility constraint, ∇·V=0, for the dispersion velocity field V. The dispersion-averaged pressure field, $P\left(r\right)=\Pi \left(r\right)+p_{f}\left(r\right)$, is the sum of the equilibrium particle osmotic pressure, Π, and a fluid-phase pressure contribution, $p_{f}$, adjusting itself such that incompressibility is fulfilled. There is a contribution to the Stokes equation proportional to dη/dϕ, operative in the inhomogeneous CP layer region.

The dispersion-averaged, stationary local particle flux, J(r), with

(11) J=ϕV−D(ϕ)∇ϕ

has an advection contribution, ϕ(r)V(r), and a diffusion contribution, −D(ϕ(r))∇ϕ(r), respectively, with the latter quantified by the gradient diffusion coefficient D(ϕ) introduced in Equation (3). Substituting this flux into the macroscopic continuity equation expressing mass conservation leads to the steady-state advection–diffusion equation,

(12) $$V\cdot \nabla \phi =\nabla \cdot \left(D(\phi )\nabla \phi \right)\;,$$

where ∂ϕ/∂t=0. Since we assume fully particle-retentive membranes, it holds that $J\cdot \hat{y}=0$ at the lumen side of the membranes.

As it is discussed in [15], in the membrane interior, the pressure gradient in the axial direction is much smaller than in the transversal direction. This allows for a transversal integration of the local Darcy equation, describing the pore-size-averaged flow inside the membrane, across the membrane thickness *h*. The result of this integration is the Darcy–Starling (boundary condition) relation [2]

(13) $$v_{w}\left(z\right)=L_{p}\left[\Delta_{T}P\left(z\right)-\Pi \left(\phi_{w}\left(z\right)\right)\right]\;,$$

for the transversal flow velocity (permeate flux), $v_{w}\left(z\right)$, at the inner membrane wall(s). The permeate flux is here defined with a positive sign. In FMS geometry, $v_{w}$ is obviously zero at the impermeable lower substrate wall. Here, $\Delta_{T}P\left(z\right)=P_{w}\left(z\right)-P^{perm}$ is the local transmembrane pressure at axial distance *z* from the inlet, and $P_{w}\left(z\right)$ and $\phi_{w}\left(z\right)$ are the lumen-side pressure and particle volume concentration at the membrane wall(s), respectively. The pressure at the permeate side, $P^{perm}$, is taken as constant.

The hydraulic permeability, $L_{p}$, of a clean membrane is given by [38,39]

(14) $$L_{p}=\left\{\begin{array}{cc} \kappa /\left(\eta_{s}R\;\ln\left(1+h/R\right)\right) & \left(\mathrm{CM}\right)\\ \kappa /\left(\eta_{s}h\right) & \left(\mathrm{FMM}/\mathrm{FMS}\right) \end{array}\right.\;,$$

where κ is the mean Darcy permeability of the membrane [40,41,42], averaged over its thickness *h*. Notice the logarithmic curvature correction for a cylindrical membrane (CM), which matters since *R* and *h* are of comparable magnitude in UF [8].

The boundary conditions at the inlet and outlet ports are, for |y|≤R,

(15) $$\begin{array}{ccc} \phi (y,z=0) & = & \phi_{b}\\ P(y,z=L) & = & P^{L} \end{array}$$

and

(16) $$\begin{array}{c} u\left(y,z=0\right)=u^{0}\left(1-\frac{y^{2}}{R^{2}}\right)\;. \end{array}$$

Here, $\phi_{b}$ is the feed concentration of particles at the inlet, and $P^{L}$ is the pressure given at the outlet. The velocity field at the inlet port (where z=0) is taken here as a fully developed parabolic flow in axial direction, so that v(y,z=0)=0. Furthermore, $u^{0}=u\left(y=0,z=0\right)$ is the axial velocity at the inlet center.

The present UF model is specified by the input (operating) parameters $\{\phi_{b},u^{0},P^{L},$$P^{perm}\}$. The pressure at the outlet is fixed to $P^{L}=1$ atm, i.e., it is taken as the atmospheric pressure. In the results presented in Section 5, the values of these parameters are selected such that there is no unwarranted axial flow exhaustion or permeate flow reversal. Conditions for the absence of these phenomena are discussed in [15].

For the given values of $\phi_{b}$, $u^{0}$, and $P^{L}=1$ atm, and instead of specifying the permeate pressure, we use alternatively as a fourth input parameter the channel-length-averaged linearized transmembrane presssure, $\langle \Delta_{T}^{\left(l\right)}P\rangle$, referred to as linearized TMP for short. The linearized TMP is the length-average of the linear axial pressure profile minus the constant permeate pressure, i.e.,

(17) $$\begin{array}{c} \Delta_{T}^{\left(l\right)}P\left(z\right)=P^{\left(l\right)}\left(z\right)-P^{perm}=P^{\left(l\right)}\left(0\right)-\left[P^{\left(l\right)}\left(0\right)-P^{L}\right]\frac{z}{L}-P^{perm}\;, \end{array}$$

associated with a Hagen–Poiseuille-type quadratic velocity field inside the CM, FMM, and FMS channels occurring for $L_{p}=0$ and $\phi_{b}=0$. The brackets denote the channel-length average,

(18) $$\begin{array}{c} \langle \left(\cdots \right)\rangle =\frac{1}{L}\int_{0}^{L}\;\;\left(\cdots \right)\;\;dz\;. \end{array}$$

The linearized TMP and the actual TMP, $\langle \Delta_{T}P\rangle$, are thus expressed as

(19) $$\begin{array}{cc} \langle \Delta_{T}^{\left(l\right)}P\rangle & =\frac{1}{2}\left[P^{\left(l\right)}\left(0\right)+P^{L}\right]-P^{perm} \end{array}$$

(20) $$\begin{array}{cc} \langle \Delta_{T}P\rangle & =\langle P\left(z\right)-P^{perm}\rangle \;, \end{array}$$

respectively. Here, P(z) is the, in general, non-linear dispersion pressure profile in UF. Moreover, it is $P^{\left(l\right)}\left(0\right)=u^{0}\lambda_{1}\eta_{s}L/R_{H}^{2}+P^{L}$, with the hydraulic radius $R_{H}=\left\{R/2,\;\;R,\;\;R\right\}$ and the dimensionless geometry coefficient $\lambda_{1}=\left\{1,\;\;2,\;\;2\right\}$ for {CM,FMM,FMS}, respectively (see Table 1). The reason for using the linearized TMP as input parameter is that for values of $L_{p}$ commonly encountered in UF, the actual TMP is practically equal to the linearized one (see [15]). The length-averaged permeate flux is related to the TMP by $\langle v_{w}\rangle =L_{p}\left[\langle \Delta_{T}P\rangle -\langle \Pi \rangle \right]$.

A useful process indicator characterizing the filtration efficiency is the solvent recovery indicator, β, given by [2,43]

(21) $$\begin{array}{c} \beta =\frac{Q^{perm}}{Q^{0}}=\frac{M\langle v_{w}\rangle }{A\;\;{\bar{u}}^{\;0}}\;. \end{array}$$

Here, ${\bar{u}}^{\;0}$ is the cross-sectional average of the inlet velocity u(y,z=0). The overline indicates the cross-sectional average,

(22) $$\begin{array}{c} \bar{\left(\cdots \right)}=\frac{1}{A}\int_{A}\left(\cdots \right)\;\;dS\;, \end{array}$$

with the constant cross-sectional area equal to $A=\pi R^{2}$ for CM, and A=RW both for FMM and FMS. Furthermore, $Q^{0}=A\;\;{\bar{u}}^{\;0}$ is the dispersion volume flow rate through the inlet cross-section, and $Q^{perm}=M\;\;\langle v_{w}\rangle$ is the permeate volume flow rate through the inner membrane area *M*. This area is equal to {2πRL,2WL,WL} for {CM,FMM,FMS}, respectively. A larger value for β reflects a larger concentration of particles at the outlet port. Notice that volume conservation implies that $Q^{0}=Q^{L}+Q^{perm}$, where $Q^{L}$ is the dispersion volume flow rate through the outlet cross section.

## 4. Boundary Layer Analysis

In this section, we generalize the modified boundary layer analysis (mBLA) method, introduced in [15] for the CM geometry, to the flat sheets systems FMM and FMS. Since the mBLA method is explained in [15], we only summarize the essentials of the method, with our focus set on the differences between the considered flat-sheet and cylindrical membrane systems.

For standard UF operating conditions, the CP layer is a thin boundary layer of characteristic thickness $\delta_{CP}\ll R$, across which ϕ is steeply decreasing, from its maximal value, $\phi_{w}\left(z\right)$, attained at the wall towards its minimal bulk value $\phi_{b}$. On introducing the smallness parameter $\epsilon_{\delta }=\delta_{CP}/R$ with $\epsilon_{\delta }\geq R/L\ll 1$, and for appropriately selected base units, the advection–diffusion equation is seen to be singularly perturbed. From a dominant balance analysis of this equation, the smallness parameter is identified as the inverse of the transversal Pèclet number, $Pe_{R}$, i.e., [15,25,44]

(23) $$\begin{array}{c} \epsilon_{\delta }=\frac{1}{Pe_{R}}=\frac{D_{0}}{R\left(L_{p}\Delta_{T}^{\left(l\right)}P\right)}\;. \end{array}$$

The transversal Pèclet number is the ratio of the diffusion time of particles, $R^{2}/D_{0}$, and the transversal flow advection time, $R/\left(L_{p}\Delta_{T}^{\left(l\right)}P\right)$, across the transversal distance *R*. It is hereby assumed that the feed concentration at the inlet is small, i.e., $\phi_{b}\ll 1$. For a significantly developed CP layer, $Pe_{R}$ is of the order $O\left[10^{2}\right]$. We describe in the following how the flow and concentration fields outside and inside the boundary layer are obtained and asymptotically matched.

### 4.1. Outer Solution

The partial differential equations determining the outer flow and concentration solutions are obtained from expanding the effective Stokes, continuity, and advection–diffusion Equations (9), (10) and (12), respectively, up to zeroth order in $\epsilon_{\delta }$. The result is [15]

(24) $$\begin{array}{cc} \frac{\partial v}{\partial y}+\frac{\partial u}{\partial z} & =-\frac{v}{y} \end{array}$$

(25) $$\begin{array}{cc} \frac{\partial P}{\partial y} & =0 \end{array}$$

(26) $$\begin{array}{cc} \frac{\partial P}{\partial z}-\frac{\partial }{\partial y}\left(\eta \left(\phi \right)\frac{\partial u}{\partial y}\right) & =\frac{\eta (\phi )}{y}\frac{\partial u}{\partial y} \end{array}$$

(27) $$\begin{array}{cc} v\frac{\partial \phi }{\partial y}+u\frac{\partial \phi }{\partial z} & =0\;. \end{array}$$

The curvature-related contributions on the right-hand side are non-zero for the CM geometry only. Using the associated boundary conditions specified in Section 3 up to the zeroth order in $\epsilon_{\delta }$, and the Darcy–Starling expression, the above set of linear differential equations is solved by separation of variables and variation of constants. Depending on the channel geometry, the outer concentration and flow solutions are $\phi \left(y,z\right)=\mathrm{const}.=\phi_{b}$ and

(28) $$\begin{array}{cc} u(y,z) & =U^{out}\left(y\right)u_{0}\left(z\right) \end{array}$$

(29) $$\begin{array}{cc} v(y,z) & =V^{out}\left(y\right)v_{w}\left(z\right) \end{array}$$

with longitudinal velocity factor

(30) $$\begin{array}{c} U^{out}\left(y\right)=1-{\left(\frac{y}{R}\right)}^{2}\;\left(\mathrm{CM}/\mathrm{FMM}/\mathrm{FMS}\right)\;, \end{array}$$

and transversal velocity factor

(31) $$\begin{array}{c} V^{out}\left(y\right)=\left\{\begin{array}{cc} 2\frac{y}{R}-{\left(\frac{y}{R}\right)}^{3} & \left(\mathrm{CM}\right)\\ \frac{1}{2}\left[3\frac{y}{R}-{\left(\frac{y}{R}\right)}^{3}\right] & \left(\mathrm{FMM}\right)\\ \frac{1}{4}\left[3\frac{y}{R}-{\left(\frac{y}{R}\right)}^{3}+2\right] & \left(\mathrm{FMS}\right) \end{array}\right.\;. \end{array}$$

To zeroth order in $\epsilon_{\delta }$, the outer velocity field is factorized in *y* and *z*. The permeate flux, $v_{w}\left(z\right),$ depends on the pressure field, P(z), through the Darcy–Starling expression, while the axial velocity at the center-line, $u_{0}\left(z\right)=u\left(0,z\right)$, is determined by the pressure according to $u_{0}\left(z\right)=-\left(\lambda_{1}R_{H}^{2}/\eta_{s}\right)dP/dz$.

The pressure, in turn, is obtained as

(32) $$\begin{array}{cc} \frac{P(z,\left[\phi_{w}\right])-P^{perm}}{\Delta_{L}^{\left(l\right)}P} & =\left(B_{+}\left[\phi_{w}\right]+g_{-}\left(z,\left[\phi_{w}\right]\right)\right)e^{K\;z/L}+\left(B_{-}\left[\phi_{w}\right]+g_{+}\left(z,\left[\phi_{w}\right]\right)\right)e^{-K\;z/L} \end{array}$$

where $\Delta_{L}^{\left(l\right)}P=P^{\left(l\right)}\left(0\right)-P^{L}$ and

(33) $$\begin{array}{cc} g_{\pm }\left(z,\left[\phi_{w}\right]\right) & =\pm \frac{K}{2L\;\Delta_{L}^{\left(l\right)}P}\int_{0}^{L}e^{\pm K\;z^{\prime }/L}\Pi \left(\phi_{w}\left(z^{\prime }\right)\right)dz^{\prime }, \end{array}$$

(34) $$\begin{array}{cc} B_{\pm }\left[\phi_{w}\right] & =\frac{1}{2\cosh(K)}\left[\frac{P^{L}-P^{perm}}{\Delta_{L}^{\left(l\right)}P}\mp \frac{1}{K}e^{\mp K}-g_{+}\left(L,\left[\phi_{w}\right]\right)e^{-K}-g_{-}\left(L,\left[\phi_{w}\right]\right)e^{K}\right]\;. \end{array}$$

We have introduced here the dimensionless effective permeability parameter, *K*, given by

(35) $$K^{2}\equiv \lambda_{1}\lambda_{2}\frac{\eta_{s}L_{p}L^{2}}{R_{H}^{3}}=\frac{\lambda_{1}\;\;M\;\;L}{{\bar{U}}^{out}\;\;A\;\;R_{H}}\cdot \frac{\kappa }{H\;\;R_{H}}\;.$$

It quantifies the overall solvent permeability of the membrane and the, in general, non-linear longitudinal pressure drop along the length of the membrane (c.f. [15]). The permeability parameter depends on the system geometry through the dimensionless coefficients $\lambda_{1}$ and $\lambda_{2}$, with $\lambda_{2}=\left\{2,\;\;3/2,\;\;3/4\right\}$ for {CM,FMM,FMS}, on the (curvature-corrected) membrane thickness given by $H=R\ln\left(1+h/R\right)$ for CM, and H=h for FMM and FMS, respectively, and on the cross-sectional average, ${\bar{U}}^{out}$, of $U^{out}\left(y\right)$ equal to 1/2 for CM and 2/3 for FMM and FMS. Table 1 summarizes the geometry-dependent quantities determining the outer solution.

For the pressure *P* and transversal velocity *v*, no distinction is required between inner and outer solutions, since to the first order in $\epsilon_{\delta }$, these quantities do not change steeply across the CP layer. Moreover, to the first order in $\epsilon_{\delta }$, the pressure is independent of *y*.

### 4.2. Inner Solution

We present next the essential steps leading to the inner boundary layer solution. As noted before, by a dominant balance analysis of the advection–diffusion equation, the smallness parameter is identified as $\epsilon_{\delta }=1/Pe_{R}$. The leading-order continuity, effective Stokes, and advection–diffusion equations determining the inner solutions are obtained as [15]

(36) $$\begin{array}{cc} 0 & =\frac{\partial v}{\partial y} \end{array}$$

(37) $$\begin{array}{cc} 0 & =\frac{\partial }{\partial y}\left(\eta \left(\phi \right)\frac{\partial u}{\partial y}\right) \end{array}$$

(38) $$\begin{array}{cc} v\frac{\partial \phi }{\partial y} & =\frac{\partial }{\partial y}\left(D\left(\phi \right)\frac{\partial \phi }{\partial y}\right)\;, \end{array}$$

respectively. The first (continuity) and second (Stokes) equation state that the transversal velocity, *v*, and the shear stress, τ=η∂u/∂y, are independent of *y* inside the CP layer, to leading order in $\epsilon_{\delta }$. Thus, $\tau =\tau_{w}\left(z\right)=\eta \left(\phi_{w}\left(z\right)\right){\left.\left(\partial u/\partial y\right)\right|}_{w}\left(z\right)$ is the shear stress at the membrane or substrate wall. Equation (38) describes the balance of transversal advection and diffusion currents. In conjunction with the boundary conditions at the membrane and substrate walls, the inner solutions for the concentration and velocity fields are obtained as

(39) $$\begin{array}{cc} \phi (y,z,\left[\phi_{w}\right]) & =\phi_{w}\left(z\right)e^{-Pe_{R}\;Y\left(y,z,\left[\phi_{w}\right]\right)} \end{array}$$

(40) $$\begin{array}{cc} u(y,z,\left[\phi_{w}\right]) & =-\tau_{w}\left(z,\left[\phi_{w}\right]\right)\int_{y}^{R}\frac{1}{\eta (\phi \left(y^{\prime },z,\left[\phi_{w}\right]\right))}dy^{\prime } \end{array}$$

(41) $$\begin{array}{cc} v(z,\left[\phi_{w}\right]) & =v_{w}\left(z,\left[\phi_{w}\right]\right)\;, \end{array}$$

where the functional dependence on the (up to this point) unknown concentration profile, $\phi_{w}\left(z\right)$, is indicated. We have introduced the dimensionless function

(42) $$Y\left(y,z,\left[\phi_{w}\right]\right)=\frac{v_{w}\left(z,\left[\phi_{w}\right]\right)}{L_{p}\Delta_{T}^{\left(l\right)}P}\frac{1}{R}\int_{y}^{R}\frac{D_{0}}{D\left(\phi (y^{\prime },z,\left[\phi_{w}\right])\right)}\;dy^{\prime }\;,$$

appearing in the inner solutions for ϕ and *u*. The transversal variable *y* is restricted to the interval [−R,R] for the FMS geometry, which lacks reflection symmetric with respect to the plane y=0. For CM and FMM, *y* is further restricted to [0,R], since, for FMM, it holds that ϕ(−y)=ϕ(y) as noticed in Figure 1.

### 4.3. Asymptotic Matching and Particle Conservation

Up to now, we determined the inner- and outer-flow solutions except for the wall concentration profile $\phi_{w}$ on which they are functionally dependent. Next, the outer limit of the inner solution is asymptotically matched to the inner limit of the outer solution, using additive and multiplicative mixing rules [45] as detailed for the CM geometry in [15]. This leads to the matched asymptotic solutions

(43) $$\begin{array}{cc} \phi (y,z,\left[\phi_{w}\right]) & =\left(\phi_{w}\left(z\right)-\phi_{b}\right)e^{-Pe_{R}\;Y\left(y,z,\left[\phi_{w}\right]\right)}+\phi_{b}\left(1-Pe_{R}\;Y\left(y,z,\left[\phi_{w}\right]\right)\;e^{-Pe_{R}\;Y\left(y,z,\left[\phi_{w}\right]\right)}\right) \end{array}$$

(44) $$\begin{array}{cc} u(y,z,\left[\phi_{w}\right]) & =u_{0}\left(z\right)\;U\left(y,z,\left[\phi_{w}\right]\right) \end{array}$$

(45) $$\begin{array}{cc} v(y,z,\left[\phi_{w}\right]) & =v_{w}\left(z\right)\;V^{out}\left(y\right)\;. \end{array}$$

The fields $P(z,\left[\phi_{w}\right])$ and $V^{out}\left(y\right)$ require no asymptotic matching and are given in Section 4.1. The longitudinal velocity factor appearing in Equation (44) for the longitudinal velocity *u* reads

(46) $$U\left(y,z,\left[\phi_{w}\right]\right)=\left(1+\frac{y}{R}\right)\frac{\int_{y}^{R}\eta^{-1}\left(\phi \left(y^{\prime },z,\left[\phi_{w}\right]\right)\right)dy^{\prime }}{\int_{0}^{R}\eta^{-1}\left(\phi \left(y^{\prime },z,\left[\phi_{w}\right]\right)\right)dy^{\prime }}\;,$$

with $U(y=0,z,\left[\phi_{w}\right])=1$. For constant viscosity, it reduces to $U\left(y,z,\left[\phi_{w}\right]\right)=U^{out}\left(y\right)$, where $U^{out}\left(y\right)$ is the parabolic profile in Equation (30).

The remaining task is to determine the particle concentration profile, $\phi_{w}\left(z\right)$, at the membrane wall. To this end, we employ, as a global condition, the cross-sectional particle-flux conservation law,

(47) $$\begin{array}{cc} \bar{J_{z}}\left(z,\left[\phi_{w}\right]\right) & =\bar{J_{z}}\left(z=0\right)\equiv {\bar{J_{z}}}^{\;0}\;, \end{array}$$

where $J_{z}=J\cdot \hat{z}$ is the longitudinal component of the particle flux in Equation (11). The overline denotes the cross-sectional average introduced in Equation (22). The above conservation law is a consequence of ∇·J=0 and of assuming a fully particle retentive membrane. We ignore here a small longitudinal diffusion flux contribution of $O[\epsilon_{\delta }^{2}]$, with the implication that $J_{z}\approx \phi \;\;u$. Notice that ${\bar{J_{z}}}^{0}$ is determined, according to the inlet boundary conditions, by $\phi_{b}$ and $u^{0}=u\left(y=0,z=0\right)$. We deviate here from our earlier mBLA work in [15] where the inlet pressure was prescribed as an inlet boundary value instead of $u^{0}$.

Using particle-flux conservation combined with a fixed-point iteration method adapted to the flat sheets FMM and FMS geometries, $\phi_{w}\left(z\right)$ is numerically determined. The concentration and flow fields follow then from substituting $\phi_{w}\left(z\right)$ into the respective matched asymptotic solutions. This constitutes our mBLA method, generalized to the FMM and FMS membrane geometries.

**Figure 5:**
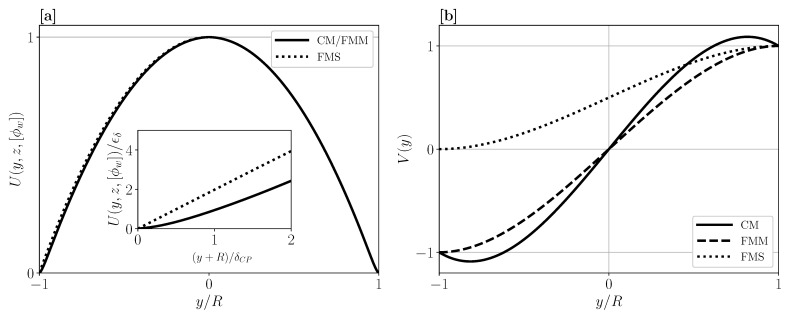
(**a**) mBLA prediction for the longitudinal velocity factor $U(y,z,\left[\phi_{w}\right])$, using $\phi_{w}=0.4$, a=10 nm, $\epsilon_{\delta }\approx 1.28\times 10^{-2}$, and $v_{w}=3.35\times 10^{-6}$ m/s. The inset shows the stretched $U/\epsilon_{\delta }$ as a function of the stretched distance from the bottom wall, $\left(y+R\right)/\delta_{CP}$. While the bottom wall is a permeable membrane for FMM, it is impermeable for FMS. (**b**) Transversal velocity factor $V\left(y\right)=V^{out}\left(y\right)$ in Equation (31) for CM, FMM, and FMS, respectively.

To analyze general features of the mBLA solution, one often introduces simplifications. Consider, for example, the longitudinal velocity factor, $U(y,z,\left[\phi_{w}\right])$ in Equation (46), entering into the matched asymptotic expression for the axial velocity *u*. In the bulk region away from the membrane wall(s), this factor practically equals the parabolic profile $1-y^{2}/R^{2}$. Differences from the parabolic profile are visible in the CP boundary layer region, provided they are enhanced by dividing $U(y,z,\left[\phi_{w}\right])$ through $\epsilon_{\delta }$. Figure 5a shows a simplified mBLA result for $U(y,z,\left[\phi_{w}\right])$, obtained by neglecting for simplicity the *z*-dependence of the matched solutions in Equations (43)–(46), and using constant $\phi_{w}\left(z\right)=0.4$, $\epsilon_{\delta }=1.28\times 10^{-2}$, a=10 nm, $\phi_{b}=0.001$, and R=0.5 mm. For the suspension transport properties D(ϕ) and η(ϕ) of Brownian hard spheres, we use the accurate expressions in Equations (3) and (5). In Figure 5a, the curves of *U* for the symmetric geometries CM and FMM are identical and practically equal to $1-y^{2}/R^{2}$ except for the boundary layer region y≈(±)R magnified in the inset. In the inset, $U\left(y,z,\left[\phi_{w}\right]\right)/\epsilon_{\delta }$ is plotted as a function of the stretched distance, $\left(y+R\right)/\delta_{CP}=\left(y/R+1\right)/\epsilon_{\delta }$, from the bottom wall (in case of FMM and FMS). Regarding the FMS geometry, a slight deviation from symmetry with respect to the y=0 plane is noticeable in the bulk region adjacent to the lower impermeable substrate wall. The inset reveals for the FMS geometry that its stretched longitudinal velocity factor near the substrate wall is larger than that for FMM, due to the absence of a CP layer at the impermeable substrate wall.

For comparison, in Figure 5b, the velocity factor $V\left(y\right)=V^{out}\left(y\right)=V^{in}\left(y\right)$ according to Equation (31) is shown for the three geometries. Note that no distinction is required between inner and outer forms of this velocity factor. Different from flat sheet geometries and owing to curvature, V(y) for CM has its peak value attained away from the membrane wall at radial distance $y=\sqrt{2/3}R$ from the center-line. While V(y=0)=0 in CM and FMM due to symmetry, the curve of V(y) in FMS is strongly asymmetric with V(y=−R)=0, which reflects the impermeability of the substrate wall.

This completes our presentation of the mBLA method for cylindrical and flat sheets membrane geometries. Table 2 summarizes the conditions for the validity of this method.

### 4.4. Remarks on the Generalized mBLA Method

Having established the generalization of the mBLA method to flat sheets geometries, a few remarks are in order here about the pros and cons of the method, its relation to other works on UF, and possible extensions to more complex dispersions.

The mBLA method for the CM geometry was shown (in [15]) to be in excellent accord with elaborate finite element calculations. While no FEM calculations are presented for the flat sheet geometries, it can be expected that the accuracy of the mBLA remains comparably good. For the CM geometry, the C++ implementation of the mBLA method is typically a thousand times faster than the according FEM calculations and it is numerically stable [15]. By using the generalized mBLA method, spatially resolved UF concentration and flux profiles are determined in addition to their spatial averages, based on theoretically precise and experimentally well-tested expressions for the dispersion properties. In case one is interested in spatial averages only, e.g., in order to compare with experimental data for the length-averaged permeate flux and TMP, a number of more simple methods are given in the literature as summarized in [2,46]. The mBLA method in its present form deals with generic CP layer effects only. Specific fouling mechanisms are not considered so far, but work on the inclusion of reversible cake layer effects is in progress. Moreover, we are currently extending the mBLA method to structured membranes as discussed in [47,48,49].

Since only the osmotic pressure and the transport properties D(ϕ) and η(ϕ) are required as input characterizing the filtered dispersion, the generalized mBLA method is readily applicable to more complex dispersions of practical relevance. These include dispersions of soft and solvent-permeable colloidal particles such as micro- and nanogels [15,31,43,50,51], and charged rigid colloidal particles, ionic microgels, and proteins [52,53,54,55]. For charged particle dispersions at lower ionic strength, the concentration dependencies of *D*, η, and Π are distinctly different from those of electrically neutral particles, with accordingly strong differences in the UF performance (see [50,51,54]).

In closing this section, we note that a key ingredient of the generalized mBLA method is a transversal advection–diffusion equation for the CP layer region. This equation is obtained from a dominant balance analysis explained in great detail in [15]. Such a dominant balance analysis was invoked also in earlier work by Denisov [25], but only for constant values of the gradient diffusion coefficient and dispersion viscosity and in conjunction with the highly approximate linear Van’t Hoff expression for the osmotic pressure. A dominant balance analysis of the advection–diffusion balance in the CP layer different from the one used in this work leads to another similarity solution for the CP layer profiles. The similarity solution was used in previous boundary layer theory studies of UF [56,57,58,59]. For the operating conditions employed in [15] and the present work, the similarity solution results, however, are in distinctly less good agreement with finite-element benchmark calculations (c.f. Figure 6 in [15]).

## 5. Results and Discussions

We quote first the employed input (operating) parameters, namely, the feed volume concentration, $\phi_{b}$, axial velocity at the inlet center, $u^{0}$, and linearized TMP, $\langle \Delta_{T}^{\left(l\right)}P\rangle$. The pressure at the outlet is fixed to $P^{L}=1$ atm. These parameters are complemented by the selected membrane and dispersion parameters including *R* and *L*, membrane hydraulic permeability, $L_{p}$, solvent viscosity, $\eta_{s}$, of water at room temperature, and particle radius *a*. While these parameters are partially varied in the presented results, as a reference set of values, we use $\phi_{b}=0.001$, R=0.5 mm, L=0.5 m, and $L_{p}=6.7\times 10^{-10}$ m/(Pa s). The selected value for $L_{p}$ is in the range of typical UF permeabilities [2], and a=3.13 nm. Reference values for $u^{0}$ depend on the membrane geometry and are selected as $\left\{6.80,\;\;5.09,\;\;5.09\right\}\times 10^{-2}$ m/s for {CM,FMM,FMS}, respectively. In all three geometries, the same value, ${\bar{u}}^{0}=3.40\times 10^{-2}$ m/s, is obtained for the cross-section averaged inlet velocity, where $u_{\mathrm{CM}}^{0}=4/3\times u_{\mathrm{FMM}/\mathrm{FMS}}^{0}$ and $u^{0}=u\left(0,0\right)$. The linearized TMP is varied from 1 kPa up to 20 kPa. The filtrated dispersion consists of mono-disperse Brownian hard spheres, suspended in water at room temperature. The particle transport properties, D(ϕ) and η(ϕ), and the osmotic pressure, Π(ϕ), are accounted for in the mBLA method using accurate analytic expressions given in Section 2.

Using the reference values for $\left\{R,L,L_{p},\eta_{s}\right\}$, the geometry-dependent reference values for the effective permeability parameter *K* defined in Equation (35) are obtained as {0.146,0.0634,0.0448} for {CM,FMM,FMS}, respectively. Since $K^{2}\ll 1$, the actual TMP, $\langle \Delta_{T}P\rangle$, is practically equal to the linearized $\mathrm{TMP}=\langle \Delta_{T}^{\left(l\right)}P\rangle$. For this reason, we do not distinguish any more in the following between actual and linearized TMP.

In Section 5.1, we discuss mBLA results for the wall concentration profile, $\phi_{w}\left(z\right)$, and for the longitudinal particle flux $J_{z}\left(y,z\right)$. For the latter quantity, both its bulk and excess parts are discussed. The TMP is not varied here, but two different particle radii a=3.13 and 10 nm are considered. In Section 5.2, the TMP, feed concentration, and cross-section averaged inlet velocity dependence of the length-averaged permeate flux, $\langle v_{w}\rangle$, and of the solvent recovery indicator β defined in Equation (21) are studied. Triggered by the results in Section 5.2, in Section 5.3, three independent parameter combinations out of the vast number of operating, membrane, and dispersion parameters are identified, which solely determine the solvent recovery indicator β.

### 5.1. CP-Layer and Longitudinal Particle Transport for Reference Conditions

**Figure 6:**
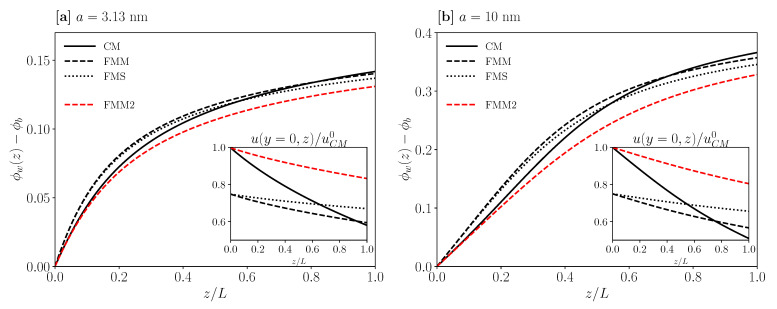
CM, FMM, and FMS concentration profiles at the membrane wall, $\phi_{w}\left(z\right)$, for a dispersion of Brownian hard spheres of radius a=3.13 nm (**a**) and a=10 nm (**b**), respectively. The insets show the according axial velocity profiles, u(y=0,z), at the channel center-line y=0, in units of the CM inlet velocity $u_{CM}^{0}=u\left(0,0\right)$. Black curves are for the same mean inlet velocity ${\bar{u}}^{0}$. Dashed (red) curves represent a second FMM system (referred to as FMM2), having a flow shear rate at the membrane inlet equal to that for the CM geometry.

For the UF geometries in Figure 6, we compare the concentration profile, $\phi_{w}\left(z\right)$, at the membrane wall for particle radii (a) a=3.13 nm and (b) a=10 nm, respectively, for fixed transversal Pèclet number $Pe_{R}=78$, and for the reference values of $L_{p}$, *R*, and *L*. Consider first Figure 6a for the concentration profile, where the reference value for the average inlet velocity, ${\bar{u}}^{0}=3.40\times 10^{-2}$ m/s, is used in all three geometries, in addition to a common TMP value of 16 kPa. The shape of the concentration profile is quite similar in the three cases, with the maximal concentration values at the outlet being distinctly smaller than the freezing transition volume concentration $\phi_{f}=0.494$ of colloidal hard spheres. The concentration profiles for FMM and FMS are nearly the same along the full channel length. Slight differences between these profiles, and the crossover of the CM with the other two profiles at axial distance z/L>0.6, can be qualitatively understood from inspecting (in the inset) the axial velocity, u(y=0,z), at the axial center-line y=0. Note that for the CM geometry $u^{0}$ is larger than for FMM and FMS, by a factor of 4/3. At axial distances similar to those for the $\phi_{w}\left(z\right)$ profile, crossovers are observed between the three curves for u(0,z). Since $du\left(0,z\right)/dz=-\left(\lambda_{2}/R_{H}\right)v_{w}\left(z\right)$, a stronger decay of u(0,z) reflects a larger transmembrane flux, $v_{w}\left(z\right)$, and thus an enhanced transport of particles towards the membrane wall.

The usage of the same value for ${\bar{u}}^{0}$ in the three geometries implies that the shear rate at the inlet of the membrane wall, ${\dot{\gamma }}^{*}=\left(du\left(y,z=0\right)/dy\right)\left(y\to 0\right)=2\;\;u^{0}/R$ (c.f. Equation (1)), is larger for CM than for FMM and FMS. This explains the initially smaller increase in $\phi_{w}\left(z\right)$ for CM. The dashed (red) curve in Figure 6a holds when for FMM the same value for the wall shear rate ${\dot{\gamma }}^{*}$ is used as for CM (FMM2 curve). While the initial slope of $\phi_{w}\left(z\right)$ at the inlet is the same for CM and FMM2, the $\phi_{w}\left(z\right)$ values for FMM2 sufficiently distant from the inlet are distinctly smaller than in the CM case. This is reflected in the axial velocity profile u(0,z) depicted in red in the inset.

A similar analysis as for Figure 6a applies to Figure 6b, for a dispersion of larger particles of radius a=10 nm, smaller TMP =5 kPa, and cross-section averaged inlet velocity ${\bar{u}}^{0}=1.11\times 10^{-2}$ m/s equal to that of CM, FMM, and FMS. The value for ${\dot{\gamma }}^{*}$ in FMM2 is equal to the one in CM. While the same general trends are encountered as in Figure 6a, there are now distinctly larger wall concentrations observed, with $\phi_{w}\left(L\right)>0.32$ at the channel outlet.

The ratio, 〈Π〉/TMP, of length-averaged transmembrane osmotic pressure and TMP equals 0.34 and 0.18 in Figure 6a,b, respectively. It reflects the strong influence of the CP layer on the permeate flux $\langle v_{w}\rangle$, exerted through the (length-averaged) Darcy–Starling law in Equation (13). Notice further that for extreme parameter values not considered here where the longitudinal pressure drop across the channel length is comparable to the TMP, the according profiles for $\phi_{w}\left(z\right)$ can be non-monotonic in *z*, with maximum concentration attained for z<L. As discussed in [15], in the context of the CM geometry, a criterion for a strictly monotonic increase in $\phi_{w}\left(z\right)$ is that $\mathrm{TMP}>3\;\;\left(P\left(0\right)-P^{L}\right)$.

**Figure 7:**
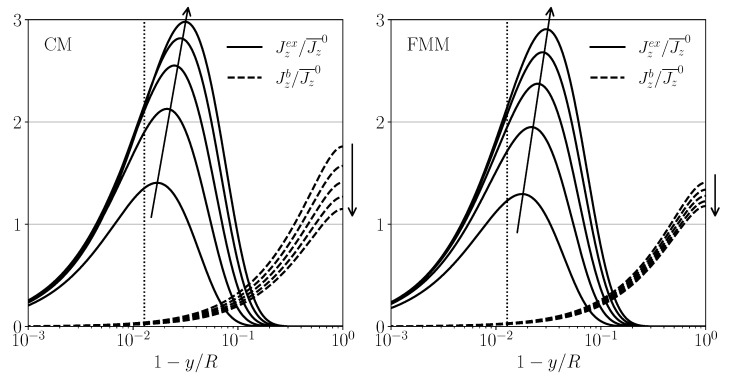
Excess and bulk axial flux parts, $J_{z}^{ex}\left(y,z\right)$ (solid lines) and $J_{z}^{b}\left(y,z\right)$ (dashed lines), plotted as functions of the reduced distance, 1−y/R, from the membrane wall, for systems CM (**left**) and FMM (**right**) using a=3.13 nm. The fluxes are given in units of ${\bar{J_{z}}}^{0}=\phi_{b}\;\;{\bar{u}}^{0}$. The arrows mark increasing values of *z*, with z/L=0.2,0.4,0.6,0.8, 1.0. The dotted vertical lines mark the distance from the membrane wall equal to $\delta_{CP}$. System parameters as in Figure 6a.

We proceed by discussing the axial particle flux, $J_{z}\left(y,z\right)$, which according to

(48) $$J_{z}\left(y,z\right)=\left(\phi \left(y,z\right)-\phi_{b}\right)\;u\left(y,z\right)+\phi_{b}\;u\left(y,z\right)\equiv J_{z}^{\;ex}\left(y,z\right)+J_{z}^{b}\left(y,z\right)\;,$$

consists of an excess flux contribution, $J_{z}^{\;ex}\left(y,z\right)$, that vanishes in the bulk region and a bulk flux contribution, $J_{z}^{b}\left(y,z\right)$. Right at the membrane wall, $J_{z}^{\;ex}\left(R,z\right)=0$ due to the employed stick boundary condition. Without a significant CP layer, $J_{z}^{\;ex}$ is practically zero. The results by the mBLA method for the excess and bulk parts of $J_{z}\left(y,z\right)$ are depicted in Figure 7 for CM and FMM, respectively, using a=3.13 nm and input parameters as in Figure 6a. The fluxes are plotted semi-logarithmically as functions of the (relative) distance, 1−y/R, from the (upper, in case of FMM) membrane wall. The FMS fluxes are not shown here since near the upper membrane, they are practically equal to those of the FMM. The excess flux is zero at the impermeable substrate wall of the FMS channel. The displayed flux profiles are given in units of the cross-sectional flux average, ${\bar{J_{z}}}^{0}=\phi_{b}\;\;{\bar{u}}^{0}$, at the inlet, which according to Equation (47), is *z*-independent. The excess axial flux (solid curves in Figure 7) increases from zero at the membrane (y=R) towards its maximal value at $y∼\delta_{CP}$. The characteristic CP layer thickness, $\delta_{CP}=R/Pe_{R}$, is marked by the vertical dotted lines. Due to a trade-off between ϕ and *u*, where the former decreases and the latter increases with increasing distance from the membrane, the maximum of $J_{z}^{ex}$ is located at a distance from the wall larger than $\delta_{CP}$, in the transition region between CP layer and bulk. The flux maximum grows with increasing distance from the inlet, and its location shifts away from the membrane wall. There is an according decrease, with increasing *z*, of the maximum values of $J_{z}^{b}$ at the channel center-line y=0.

According to Figure 7, the excess fluxes in CM are similar to those in FMM, which can be attributed to the fact that curvature effects in the thin CP layer of the CM geometry are negligible. In contrast, there are pronounced differences observed for the maximal bulk fluxes of $J_{z}^{b}\left(y=0,z\right)=\phi_{b}\;\;u\left(0,z\right)$, reflected in the likewise different behavior of u(0,z) for CM and FMM, seen in the inset of Figure 6a.

**Figure 8:**
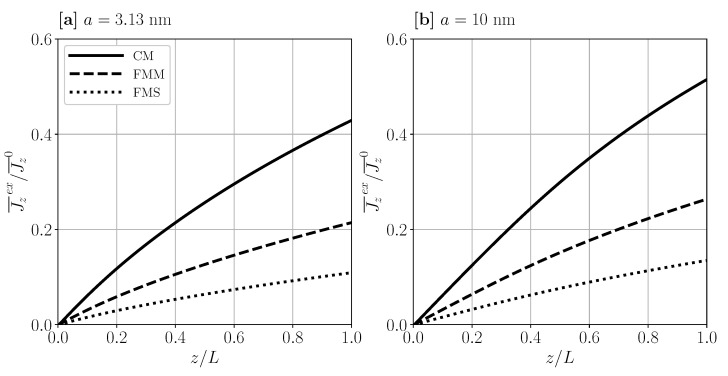
Cross-section averaged axial excess particle-flux, ${\bar{J_{z}}}^{\;ex}\left(z\right)$, in units of ${\bar{J_{z}}}^{0}$, as function of reduced axial distance, z/L, from the inlet. Two dispersions with particle radius a=3.13 nm (**a**) and a=10 nm (**b**) are considered for input parameters as in Figure 6a,b, respectively.

To elucidate differences in the axial particles transport between CM and the flat sheets FMM and FMS geometries, in Figure 8, the cross-section averaged axial excess flux, ${\bar{J_{z}}}^{\;ex}\left(z\right)$, is shown for a=3.13 nm and a=10 nm, respectively. Like $\phi_{w}\left(z\right)$, the average flux increases monotonically from 0 at the inlet towards its maximal value at the outlet where the CP layer becomes most pronounced. In both panels of Figure 8, where the same value for ${\bar{u}}^{0}$ is used, the excess flux for CM is twice as large as the one for FMM. To understand this, notice that the cross-section averaged axial bulk flux follows, from the sum rule expressing particle flux conservation and our matched asymptotic solution, as

(49) $${\bar{J_{z}}}^{b}\left(z\right)/{\bar{J_{z}}}^{0}=1-{\bar{J_{z}}}^{ex}\left(z\right)/{\bar{J_{z}}}^{0}=\frac{M}{A\;\;{\bar{u}}^{0}}\frac{1}{L}\int_{0}^{z}\;\;dz^{\prime }\;\;v_{w}\left(z^{\prime },\left[\phi_{w}\right]\right)+O\left[\epsilon_{\delta }\right]\;.$$

Recall next from Table 1 that the ratio, M/A, of membrane area *M* and cross-section area *A* is twice as large for CM as for FMM. One further observes in Figure 8 that ${\left.{\bar{J_{z}}}^{ex}\left(z\right)\right|}_{FMM}\approx 2\times {\left.{\bar{J_{z}}}^{ex}\left(z\right)\right|}_{FMS}$, which arises since the value for M/A in FMS is half that of FMM, while $v_{w}\left(z\right)$ is practically the same in both cases. In comparing Figure 8a,b, one notices, for operating conditions as those in Figure 6a,b, that the normalized excess flux curves in (b) are larger than the according ones in (a). In general, there is an intermingled influence of *D*, η, and Π on the UF behavior when the particle size is varied.

### 5.2. TMP, Feed Concentration, and Velocity Effects on Global Indicators

Having discussed the local profiles $\phi_{w}\left(z\right)$ and $J_{z}\left(y,z\right)$, we assess next the influence of varying TMP and feed concentration on properties that globally indicate the UF performance. Two global indicators of particular importance are the length-averaged permeate flux, $\langle v_{w}\rangle =L_{p}\left(\mathrm{TMP}-\langle \Pi \rangle \right)$, and the solvent recovery indicator β≤1 introduced in Equation (21). The latter indicator gives the fraction of initial dispersion volume, recovered as pure solvent in the permeate compartment. It is related to another indicator, $\alpha ={\bar{\phi }}^{L}/\phi_{b}\geq 1$, defined as the ratio of cross-section averaged concentration at the outlet and the (cross-section averaged) feed concentration $\phi_{b}$. Owing to particle flux and dispersion volume conservation valid for an ideally particle retentive membrane, the concentration factor α is related to β simply by [43]

(50) $$\alpha =\frac{1}{1-\beta }\;.$$

One generally observes that $\langle v_{w}\rangle$ increases monotonically with increasing TMP. Considering Equations (21) and (50), this implies that both β and α are monotonically increasing with increasing TMP.

**Figure 9:**
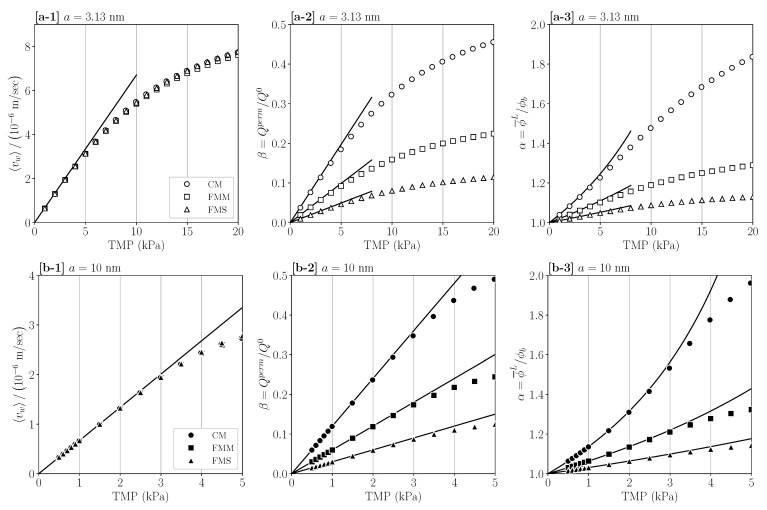
(**a-1**,**b-1**): TMP dependence of average permeate flux $\langle v_{w}\rangle$, solvent recovery indicator β, and concentration factor α for particle radius a=3.13 nm (panels **a-1**–**a-3**) and a=10 nm (panels **b-1**–**b-3**), respectively. Symbols are mBLA results, and solid lines are pure solvent predictions. Input parameters except for TMP are as in Figure 6.

Our central aim is to identify dimensionless combinations of input parameters characterizing uniquely the global process indicators, independent of the considered geometries. As a prerequisite, in Figure 9, we analyze the process indicators $\langle v_{w}\rangle$, α, and β as functions of TMP, for input parameters selected otherwise as in Figure 6a,b. The symbols are predictions by the mBLA method. Regarding the average permeate flux depicted in panels (a-1) and (b-1), the mBLA results for $\langle v_{w}\rangle$ are nearly coincidental, even for the largest TMP considered. The straight solid lines depict the pure-solvent form $\langle v_{w}^{0}\rangle =L_{p}\;\;\mathrm{TMP}$, for the same hydraulic permeability $L_{p}$ used in both figure panels. The influence of the CP layer (osmotic pressure) buildup on the permeate flux is noticeable at larger TMP where $\langle v_{w}\rangle$ increased sub-linearly. For particle radius 10 nm, a smaller TMP interval is depicted in the figure since cake formation by freezing is observed already for TMP≳7 kPa.

The straight lines in panels (a-2) and (b-2) of Figure 9 are the pure-solvent prediction, $\beta^{0}$, equal to β at zero feed concentration $\phi_{b}=0$. Explicitly,

(51) $$\beta^{0}=\left(\frac{M}{A}\frac{L_{p}}{{\bar{u}}^{0}}\right)\;\;\mathrm{TMP}\;,$$

where *M* is the lumen-side membrane surface area, and *A* is the area of the inner cross-section. As noticed from Equation (51), $\beta^{0}$ has a geometry-dependent factor, M/A, of values listed in Table 1, and it includes the cross-section averaged inlet velocity ${\bar{u}}^{0}$, which has different values in panels (a) and (b), respectively. At small TMP, the mBLA curves for β overlap with the pure-solvent lines of $\beta^{0}$, since the osmotic pressure contribution is here negligible. For comparison, panels (a-3) and (b-3) show the associated concentration factor α. Solid lines represent $\alpha^{0}=1/\left(1-\beta^{0}\right)$, which quantifies the initial increase in α with increasing TMP. Notice that, according to Equation (51), both $\beta^{0}$ and $\alpha^{0}$ are solely dependent on given input parameters. According to Table 1, it holds that $\beta_{\mathrm{CM}}^{0}:\beta_{\mathrm{FMM}}^{0}:\beta_{\mathrm{FMS}}^{0}$ = 4:2:1 for equal TMP.

**Figure 10:**
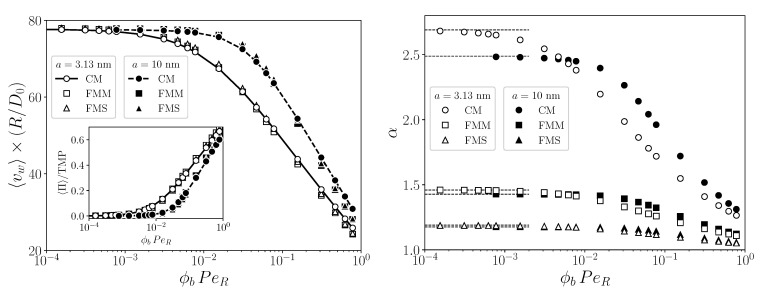
Reduced average permeate flux, $\langle v_{w}\rangle \left(R/D_{0}\right)$, (**left**) and concentration factor, α, (**right**) versus feed concentration, $\phi_{b}$, for fixed $Pe_{R}\approx 78$. Input parameters are as in Figure 6, except for $\phi_{b}$, which is varied. Open symbols are for a=3.13 nm and filled symbols for a=10 nm. The inset in the left panel shows the pressure ratio 〈Π〉/TMP. The plateau values, $\alpha^{0}$, of α are marked in the right panel by the horizontal dashed line segments.

The effect of a varying radius *a* on the length-averaged reduced permeate flux and on α is analyzed in Figure 10 (left) and (right), respectively. The permeate flux is rendered non-dimensional by multiplication with $R/D_{0}$, and it is plotted versus $\phi_{b}$ multiplied by $Pe_{R}\approx 78$, i.e., the selected value for the transversal Pèclet number. The multiplication of $\phi_{b}$ by $Pe_{R}$ is used here to highlight that $\phi_{b}\;\;Pe_{R}<1$, an inequality required for the validity of the mBLA method (c.f. Table 2). Except for $\phi_{b}$, all input parameters are as in Figure 6a,b, respectively. Regarding Figure 10 (left), one notices that $\langle v_{w}\rangle \left(R/D_{0}\right)\to Pe_{R}$ for $\phi_{b}\to 0$. According to the inset, the initial plateau value of the reduced flux, equal to $Pe_{R}$, is mirrored by the plateau value 〈Π〉/TMP≈0. With further increasing $\phi_{b}$, the accordingly increasing Π causes a decline in the reduced permeate flux linear in the logarithm of $\phi_{b}$, which was also observed in typical UF experiments [9]. For fixed *a*, the reduced flux is nearly the same for the considered geometries, except when $\phi_{b}\;\;Pe_{R}$ is close to one. The mBLA results for α as a function of $\phi_{b}$ are displayed in Figure 10 (right). The concentration factor decreases with increasing $\phi_{b}$, and it is distinctly larger for the CM geometry. One notices further in Figure 9(a-1,b-1) that different channel geometries have little influence on the permeate flux. Figure 10 (right) is useful for re-circulation setups where the concentrated dispersion, collected at the outlet port, is fed back at the inlet port.

**Figure 11:**
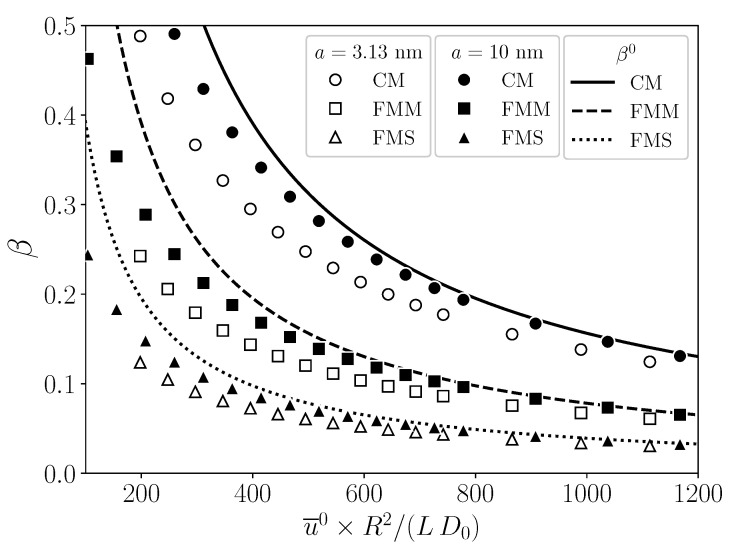
Solvent recovery indicator, β, as function of the cross-section averaged inlet velocity ${\bar{u}}^{0}$ in units of $LD_{0}/R^{2}$. Here, ${\bar{u}}^{0}$ is varied for a fixed TMP=16 kPa for a=3.13 nm, and 5 kPa for a=10 nm. The feed concentration is $\phi_{b}=0.001$. Remaining input parameters as in Figure 6a,b. Open (filled) symbols are mBLA results for a=3.13 nm (a=10 nm). Solid, dashed, and dotted lines represent the pure solvent values, $\beta^{0}$, for the respective geometries as indicated.

The dependence of β on the cross-section averaged axial inlet velocity, ${\bar{u}}^{0}$, is shown in Figure 11, for the three geometries and for two particle sizes. The velocity is made non-dimensional by multiplication with the axial velocity unit $LD_{0}/R^{2}$. With increasingly large inlet velocity, the mBLA data for β (symbols) converge towards the pure solvent prediction $\beta^{0}$ (lines). This convergence arises since the influence of the CP layer ceases with increasing axial flow velocity.

### 5.3. Universal Behavior of Global UF Indicators

In Section 5.2, we analyzed the global UF indicators α, β, and $\langle v_{w}\rangle \left(R/D_{0}\right)$, as functions of TMP, $\phi_{b}$, *a*, and ${\bar{u}}^{0}$, respectively, with the other input parameters kept constant. It was shown that the indicators are significantly affected by changes in these parameters and in the membrane. The indicators depend in addition on *R*, *L*, and $L_{p}$, which characterize the membrane geometry and hydraulic permeability, respectively.

Since the UF process depends on a vast number of input and membrane geometry parameters, it is important to identify a minimal set of dimensionless combinations of input parameters fully characterizing the UF indicators. By extensive parameter variation studies based on the mBLA method combining numerical efficiency with high accuracy, and by using a simplified mBLA solution described in Appendix A, we succeeded in identifying three independent dimensionless combinations of input parameters, termed variables in the following, on which β is solely dependent. The first of these independent variables is the transversal Pèclet number, $Pe_{R}$, introduced in Equation (23) as the ratio of diffusion and flow advection times for the particle transported across the transversal distance *R*. The remaining two variables are identified as

(52) $$\begin{array}{ccc} \frac{\beta^{0}}{Pe_{R}} & = & \frac{MD_{0}}{A\;\;R\;\;{\bar{u}}^{0}} \end{array}$$

(53) $$\begin{array}{ccc} \frac{\gamma^{0}}{Pe_{R}} & = & \left(\frac{9\;\;c}{2}\frac{\phi_{b}L_{p}\eta_{s}R}{a^{2}}\right)\frac{\beta^{0}}{Pe_{R}}\;\;\;. \end{array}$$

Here, $\beta^{0}$ is the pure solvent recovery indicator introduced in Equation (51) where its linear dependence on TMP was emphasized. While $\beta^{0}$ is independent of dispersion properties, these are accounted for by $\gamma^{0}$ and $Pe_{R}$. The third variable, $\gamma^{0}$, includes a geometry-dependent factor c={1/8,1/3,2/3} for {CM,FMM,FMS}, respectively. Moreover, it depends on the feed concentration $\phi_{b}$, particle radius *a*, hydraulic membrane permeability $L_{p}$, solvent viscosity $\eta_{s}$, and the inner channel half-height *R*. The appearance and the form of $\gamma^{0}$ are motivated by a simplified mBLA calculation of $\langle v_{w}\rangle \;R/D_{0}$ presented in Appendix A.

On expressing β in terms of the three variables, we find that

(54) $$\begin{array}{c} \beta \approx F\left(Pe_{R},\frac{\beta^{0}}{Pe_{R}},\;\;\frac{\gamma^{0}}{Pe_{R}}\right)\; \end{array}$$

is valid to good accuracy in the assessed parameter space region. Here, *F* is a function depending on the three variables only.

**Figure 12:**
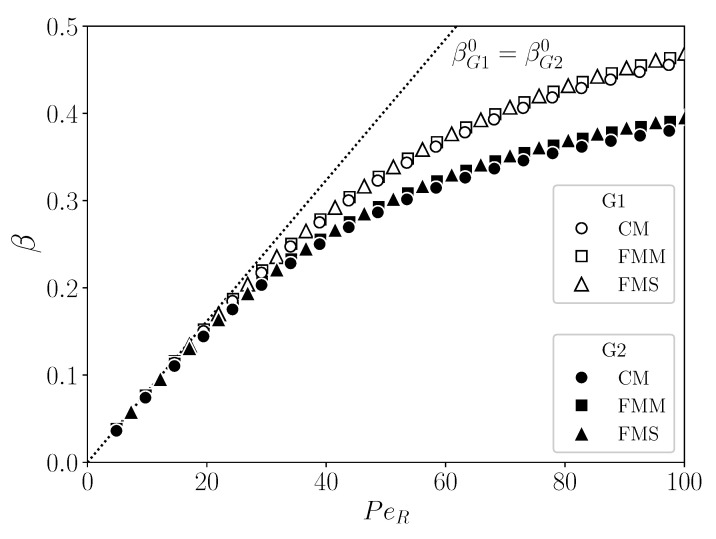
Solvent recovery indicator, β, as a function of $Pe_{R}$, for ${\left(\beta^{0}/Pe_{R}\right)}_{G1,\;G2}\approx 8.08\times 10^{-3}$, and ${\left(\gamma^{0}/Pe_{R}\right)}_{G1}\approx 1.55\times 10^{-4}$ (list G1 in Table 3: open symbols) and ${\left(\gamma^{0}/Pe_{R}\right)}_{G2}\approx 3.10\times 10^{-4}$ (list G2: filled symbols), respectively. Identical membrane properties are used for CM, FMM, and FMS. The employed operating parameters are listed in rows G1 and G2 of Table 3.

The universal behavior of β in terms of the three variables is exemplified in Figure 12, where it is plotted as a function of $Pe_{R}$, for values of the duplet $\left(\beta^{0}/Pe_{R},\;\;\gamma^{0}/Pe_{R}\right)$ given by the two sets $G1=\left(8.08\times 10^{-3},\;1.55\times 10^{-4}\right)$ (open symbols) and $G2=(8.08\times 10^{-3},$ $3.10\times 10^{-4})$ (filled symbols), respectively. Within each set, the mBLA data for β coincide practically for all $Pe_{R}$ and for the three geometries CM, FMM, and FMS. The two curves $F(Pe_{R};G1)$ and $F(Pe_{R};G2)$ traced out by the mBLA data for β merge at small $Pe_{R}$ with the straight dotted line for $\beta^{0}$ determined by Equation (52). The two sets G1 and G2 for $\left(\beta^{0}/Pe_{R},\;\;\gamma^{0}/Pe_{R}\right)$ share the same value $8.08\times 10^{-3}$ for $\beta^{0}/Pe_{R}$. The input parameters for the curves G1 and G2 in Figure 12 are listed in the rows G1 and G2 of Table 3. Notice from Figure 12 that, for given (non-small) $Pe_{R}$ and $\beta^{0}$, a larger value of $\gamma^{0}$ causes a smaller values of β due to the lowered permeate flux. According to Equation (53), $\gamma^{0}$ increases for increasing $\phi_{b}$ and $L_{p}$, and for decreasing particle radius *a*.

**Figure 13:**
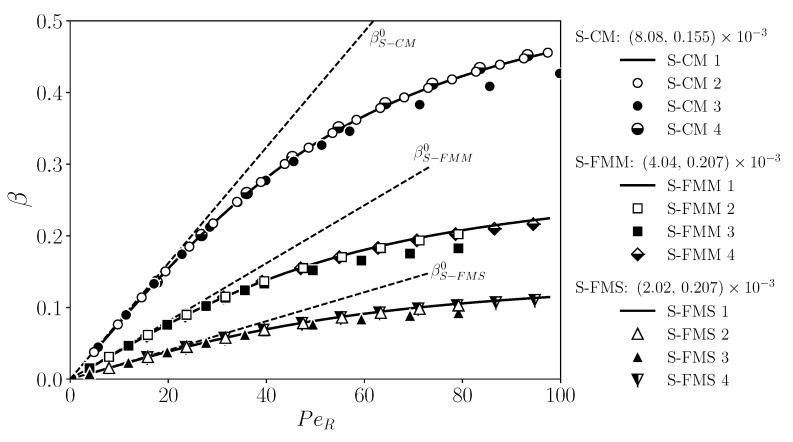
Solvent recovery indicator, β, as a function of $Pe_{R}$ for three different variable sets $\left(\beta^{0}/Pe_{R},\gamma^{0}/Pe_{R}\right)$ given in the figure for the respective CM, FMM, and FMS geometries. Symbols are mBLA results for β based on the according input parameter lists in Table 3. See text for details.

In rows G1 and G2 of Table 3, the membrane parameters *R*, *L*, and $L_{p}$ are kept constant. To illustrate that changes in these parameters are likewise compatible with Equation (54), in Figure 13, β is shown as a function of $Pe_{R}$, for three sets of the duplet $\left(\beta^{0}/Pe_{R},\;\;\gamma^{0}/Pe_{R}\right)$ given by $S-\mathrm{CM}=\left(8.08\times 10^{-3},\;1.55\times 10^{-4}\right)$ (circles), $S-\mathrm{FMM}=\left(4.04\times 10^{-3},\;2.07\times 10^{-4}\right)$ (squares), and $S-\mathrm{FMS}=\left(2.02\times 10^{-3},\;2.07\times 10^{-4}\right)$ (triangles). These three sets are not taken out of the blue but are precisely the values for $\beta^{0}/Pe_{R}$ and $\gamma^{0}/Pe_{R}$ of the reference system introduced in Section 5 for CM, FMM, and FMS, respectively. For each geometry with its according set for $\left(\beta^{0}/Pe_{R},\;\;\gamma^{0}/Pe_{R}\right)$, four different lists of input parameters are given in Table 3, e.g., rows CM 1-4 for the CM geometry. Rows CM 1, FMM 1, and FMS 1 include the reference system input data for CM, FMM, and FMS, respectively. Different values for the membrane properties are used for the three geometries. In lists CM 2-4, e.g., the thickness *h* of the membrane is specified instead of $L_{p}$, by assuming the same mean Darcy permeability, κ, as for the reference system with CM geometry. The hydraulic permeability follows then from Equation (14). According to Figure 13, β traces out three master curves for S-CM (top), S-FMM (middle), and S-FMS (bottom), respectively, where the according duplet values for $\left(\beta^{0}/Pe_{R},\;\;\gamma^{0}/Pe_{R}\right)$ are noted in the figure. The solid curves for β are obtained using reference system data listed in rows CM 1, FMM 1, and FMS 1 of Table 3. The dashed straight lines are the pure solvent predictions $\beta^{0}$ for β. It holds that $\beta_{S-\mathrm{CM}}^{0}>\beta_{S-\mathrm{FMM}}^{0}>\beta_{S-\mathrm{FMS}}^{0}$ (see also Figure 9(a-2)). The mBLA data for β in Figure 13 nicely reconfirm the validity of Equation (54).

The universal dependence of β on the three variables $Pe_{R}$, $\beta^{0}/Pe_{R}$, and $\gamma^{0}/Pe_{R}$ in Equation (54) is inherited by the concentration factor, α, and by the reduced permeate flux, $\langle v_{w}\rangle \left(R/D_{0}\right)$, since these are related to α by

(55) $$\begin{array}{c} \beta =\frac{1}{1-\alpha }=\left(\langle v_{w}\rangle \frac{R}{D_{0}}\right)\frac{\beta^{0}}{Pe_{R}}\;. \end{array}$$

The independent variables on which β, α, and the reduced permeate flux are solely dependent are identified using a simplified mBLA calculation where the gradient diffusion coefficient and viscosity are approximated by their infinite dilution values, and the osmotic pressure is approximated by the linear van’t Hoff expression. Details of this calculation are given in Appendix A. The result of the Appendix is the simplified mBLA expression

(56) $$\begin{array}{c} \langle v_{w}\rangle \frac{R}{D_{0}}\approx {\left(\frac{\beta^{0}}{Pe_{R}}+\frac{\gamma^{0}}{Pe_{R}}g\right)}^{-1}\;. \end{array}$$

Here, *g* is a complicated function given in Equation (A6) of the Appendix A, which depends on $Pe_{R}$ and other input parameters, and implicitly also on the reduced permeate flux. Thus, a fixed-point iteration solver is required also for the simplified mBLA expression in Equation (56). Note that Equation (56) holds for larger values of $Pe_{R}$ only, and different from the full mBLA solution, it violates to some extent the sole dependence on β on the three variables in Equation (54). The virtue of Equation (56) is that it suggests that $Pe_{R}$, $\beta^{0}/Pe_{R}$, and $\gamma^{0}/Pe_{R}$ are the reduced set of independent variables and that it provides, in addition, the expression for $\gamma^{0}$ in Equation (53).

## 6. Conclusions

We generalized the mBLA method for predicting local UF concentration and flow profiles, and global process indicators, from the cylindrical membrane geometry to flat sheets geometries with two and one membrane sheet, respectively. The semi-analytic form of the method makes it well suited for identifying general trends and unifying features, which are tasks that require extensive variations of different input parameters. Importantly, the semi-analytic mBLA method gives physical insight into the functional form of concentration and flow profiles and of global process indicators.

For simplicity, we considered dispersions of mono-disperse Brownian hard spheres, using accurate analytic expressions for the transport properties and osmotic pressure, as input to the generalized mBLA method. We elaborated on the effects of the three geometries on local concentration and particle flux profiles, by revealing significant differences in the average particle fluxes. For varying operating conditions, we analyzed the influence of the different geometries on global UF efficiency indicators including β, concentration factor α, and length-averaged permeate flux $\langle v_{w}\rangle$. We showed that the influence of different geometries is particularly pronounced for these indicators.

Our key result is that the intricate and intermingled dependencies of the UF indicators on a vast number of input parameters and the membrane geometry can be grouped together into only three independent variables: $Pe_{R}$, $\beta^{0}/Pe_{R}$, and $\gamma^{0}/Pe_{R}$. The semi-analytic form and numerical efficiency of the mBLA method allowed us to identify these dimensionless variables and to show that β is uniquely determined by them. The simple three-variables dependency carries over to related global indicators, and it can be of help in the design, optimization, and scale-up of UF setups.

We expect that a description of β along the lines of Equation (54) holds also for more complex dispersions such as solvent-permeable hard spheres and non-ionic nanogels, based on our experience gained for these systems [15,31,43]. It is likely that additional independent variables (parameter groups) come into play for these systems on which β is depending on. The generalized mBLA method can be used to identify these additional variables. In dealing with UF and particle size polydispersity, partially retentive membranes and fouling mechanisms are also of importance. These features were not accounted for in the present work focusing on generic CP layer and geometry effects. Work on extensions of the mBLA method to account for cake-layer effects and polydispersity is in progress.

## Figures and Tables

**Table 1:** Summary of geometry-dependent quantities characterizing the outer solution. Notice that $\lambda_{2}=R_{H}M/\left(LA{\bar{U}}^{out}\right)$. The effective permeability parameter, *K*, is given in units of $K^{*}=\sqrt{\eta_{s}L_{p}L^{2}/R^{3}}$ so that $K/K^{*}=\sqrt{\lambda_{1}\lambda_{2}{\left(R/R_{H}\right)}^{3}}$.

Membrane Geometry	Transversal Velocity Boundary Condition	$\lambda_{1}$	$\lambda_{2}$	$R_{H}$	${\bar{U}}^{\mathrm{out}}$	$\frac{M}{A}$	$\frac{K}{K^{*}}$	*H*	$V^{\mathrm{out}}\left(y\right)$
CM	$\;\;v\left(R,z\right)=v_{w}\left(z\right)$	1	2	$\frac{R}{2}$	$\frac{1}{2}$	$\frac{2L}{R}$	4	$R\ln\left(1+\frac{h}{R}\right)$	$2\frac{y}{R}-{\left(\frac{y}{R}\right)}^{3}$
FMM	$\begin{array}{cc} v(R,z) & =v_{w}\left(z\right)\\ v(-R,z) & =-v_{w}\left(z\right) \end{array}$	2	$\frac{3}{2}$	*R*	$\frac{2}{3}$	$\frac{L}{R}$	$\sqrt{3}$	*h*	$\frac{1}{2}\left[3\frac{y}{R}-{\left(\frac{y}{R}\right)}^{3}\right]$
FMS	$\begin{array}{cc} v(R,z) & =v_{w}\left(z\right)\\ v(-R,z) & =0 \end{array}$	2	$\frac{3}{4}$	*R*	$\frac{2}{3}$	$\frac{L}{2R}$	$\sqrt{\frac{3}{2}}$	*h*	$\frac{1}{4}\left[3\frac{y}{R}-{\left(\frac{y}{R}\right)}^{3}+2\right]$

**Table 2:** Summary of conditions for the validity of the mBLA method. The Reynolds number associated with the channel flow was $Re=4\;\;R_{H}\;{\bar{u}}^{0}\rho /\eta$, where ρ is the dispersion mass density, η the effective dispersion viscosity, ${\bar{u}}^{0}$ the cross-section averaged inlet velocity, and $R_{H}$ the hydraulic channel radius. The solvent recovery indicator, β, is defined in Equation (21).

Conditions	Remarks
$\;Pe_{a}\leq 0.1$	Strong Brownian motion
R/L≪1	Small aspect ratio. Note that $\epsilon_{\delta }\geq R/L$
Re≲2000	Condition for laminar (non-turbulent) flow
ReR/L≪1	No inertial flow effects on length scale *L*
$\;\;\beta \geq O\left[\epsilon_{\delta }\right]$	Condition for significant permeability effects
$\;\phi_{b}\ll 1$	Small feed concentration (i.e., $\phi_{b}Pe_{R}<1$)

**Table 3:** Input parameters used for the mBLA results for the solvent recovery indicator β depicted in Figure 12 and Figure 13, respectively, as indicated. Fixed parameters are particle radius a=3.13 nm and mean Darcy permeability $\kappa =1.36\times 10^{16}\;\;m^{2}$. The employed values for the reference system cross-section averaged inlet velocity and hydraulic permeability are ${\bar{u}}_{REF}^{0}=3.40\times 10^{-2}$ and $L_{p,REF}=6.7\times 10^{-10}$ m/(Pa s), respectively.

**Data** **(Figure 12)**	***R*** **(mm)**	***L*** **(m)**	h/R	$L_{p}/L_{p,\mathrm{REF}}$ **CM, FMM/FMS**	$\phi_{b}/10^{-3}$ **CM, FMM, FMS**	${\bar{u}}^{0}/{\bar{u}}_{\mathrm{REF}}^{0}$ **CM, FMM, FMS**	$\left(\beta^{0}/{\mathrm{Pe}}_{R}\right)/10^{-3}$	$\left(\gamma^{0}/{\mathrm{Pe}}_{R}\right)/10^{-4}$
G1	0.5	0.5	-	1.00, 1.00	1.00, 0.375, 0.186	1.00, 0.50, 0.25	8.08	1.55
G2	0.5	0.5	-	1.00, 1.00	2.00, 0.750, 0.375	1.00, 0.50, 0.25	8.08	3.10
**(Figure 13)**	R **(mm)**	L **(m)**	h/R	$L_{p}/L_{p,\mathrm{REF}}$	$\phi_{b}/10^{-3}$	${\bar{u}}^{0}/{\bar{u}}_{\mathrm{REF}}^{0}$	$\left(\beta^{0}/{\mathrm{Pe}}_{R}\right)/10^{-3}$	$\left(\gamma^{0}/{\mathrm{Pe}}_{R}\right)/10^{-4}$
CM-1	0.5	0.5	-	1.00	1.00	1.00	8.08	1.55
CM-2	0.5	0.5	0.5	1.00	1.00	1.00	8.08	1.55
CM-3	0.5	0.5	1	0.59	1.69	1.00	8.08	1.55
CM-4	0.25	0.5	0.5	2.00	1.00	4.00	8.08	1.55
FMM-1	0.5	0.5	-	1.00	1.00	1.00	4.04	2.07
FMM-2	0.5	0.5	0.5	0.81	1.24	1.00	4.04	2.08
FMM-3	0.5	0.5	1	0.41	2.44	1.00	4.04	2.07
FMM-4	0.25	0.5	0.5	1.62	1.23	4.00	4.04	2.07
FMS-1	0.5	0.5	-	1.00	1.00	1.00	2.02	2.07
FMS-2	0.5	0.5	0.5	0.81	1.24	1.00	2.02	2.08
FMS-3	0.5	0.5	1	0.41	2.44	1.00	2.02	2.07
FMS-4	0.25	0.5	0.5	1.62	1.23	4.00	2.02	2.07

## Data Availability

The data supporting the findings of this study are available from the corresponding author upon reasonable request. Our Python code for the mBLA method is freely avaliable at [60].

## Abbreviations

CP	concentration-polarization
CM	cylindrical membrane
FMM	flat sheet membranes (top and bottom)
FMS	flat sheet membrane (top)/substrate (bottom)
mBLA	modified boundary layer analysis (method)
TMP	transmembrane pressure
UF	ultrafiltration

## List of Symbols (SI Units)

$Pe_{a}$	single-particle shear-Pèclet number
$Pe_{R}$	transversal Pèclet number
α	concentration factor
β	solvent-recovery indicator
$\alpha^{0}$, $\beta^{0}$	the infinite dilution value of α, β
$\gamma^{0}$	third dimensionless variable characterizing β
$\epsilon_{\delta }=1/Pe_{R}$	perturbation parameter
$\delta_{CP}$	characteristic thickness of CP layer (m)
*R*, *L*, *W*	half-height, axial length, width of channel (m)
$R_{H}$	hydraulic radius (m)
*h*, *H*	membrane thickness, curvature-corrected thickness (m)
*A*, *M*	channel cross-section, membrane surface area (m^2^)
*y*, *z*	transversal, longitudinal coordinate (m)
V	dispersion-averaged velocity (m/s)
*v*, *u*	transversal, longitudinal velocity (m/s)
$u^{0}$	axial velocity at center of inlet (m/s)
$U^{out}$	longitudinal velocity factor of outer solution
*U*	asymptotically matched longitudinal velocity factor
$v_{w}$	permeate flux (m/s)
$V^{out}$	transversal velocity factor
${\dot{\gamma }}^{*}$	shear rate at inlet of membrane wall (1/s)
$\tau_{w}$	shear stress at membrane wall (Pa)
*P*	dispersion-averaged pressure (Pa)
$P^{perm}$, $P^{L}$	pressure at permeate side, at outlet port (Pa)
$\langle \Delta_{T}P\rangle$	length-averaged transmembrane pressure (Pa)
$\langle \Delta_{T}^{\left(l\right)}P\rangle$	length-averaged, linearized transmembrane pressure (Pa)
Π	particles osmotic pressure (Pa)
ρ	dispersion mass density (kg/m^3^)
*n*	particle number density (1/m^3^)
*a*	radius of hard spheres (m)
$V_{a}$	particle volume (m^3^)
ϕ	particle volume fraction
$\phi_{b}$, $\phi_{w}$	particle volume fraction at inlet (feed), at membrane wall
η, $\eta_{s}$	suspension-, solvent viscosity (Pa s)
*D*	gradient diffusion coefficient (m^2^)/s)
$D_{0}$	Stokes–Einstein diffusion coefficient (m^2^)/s)
κ	mean Darcy permeability (m^2^)
$L_{p}$	hydraulic permeability of clean membrane (m/(Pa s))
*K*	dimensionless effective permeability parameter
$Q^{0}$, $Q^{perm}$, $Q^{L}$	volume flow rate through inlet, membrane, outlet (m^3^)/s)
$J_{z}$, $J_{z}^{ex}$, $J_{z}^{b}$	longitudinal particle-flux, excess part, bulk part (m/s)
$\langle \left(\cdots \right)\rangle$	length-average of $\left(\cdots \right)$ (c.f. Equation (18))
$\bar{\left(\cdots \right)}$	cross-section average of $\left(\cdots \right)$ (c.f. Equation (22))

## Appendix A. Simplified mBLA Method

We present here the derivation of the simplified mBLA expression for the reduced length-averaged permeate flux, $\langle v_{w}\rangle \;\;R/D_{0}$, given in Equation (56) of Section 5.3.

On approximating the gradient diffusion coefficient, D(ϕ), and the dispersion viscosity, η(ϕ), by their infinite dilution values $D_{0}$ and $\eta_{s}$, respectively, the mBLA expression for the concentration profile at the inner membrane wall, $\phi_{w}\left(z\right)$, is reduced to

(A1) $$\phi_{w}\left(z\right)-\phi_{b}\approx c\;\phi_{b}\;Pe_{R}^{2}\;\beta^{0}\frac{{\left(v_{w}\left(z,\left[\phi_{w}\right]\right)/v^{*}\right)}^{2}}{u_{0}\left(z,\left[\phi_{w}\right]\right)/u^{0}}\frac{1}{L}\int_{0}^{z}\left(v_{w}\left(z^{\prime },\left[\phi_{w}\right]\right)/v^{*}\right)dz^{\prime }\;.$$

Here, $v^{*}=L_{P}\langle \Delta_{T}P\rangle$, and *c* is a geometry-dependent factor equal to {1/8,1/3,2/3} for the CM, FMM, and FMS geometries, respectively. The functional dependence of $v_{w}\left(z\right)$ and $u_{0}\left(z\right)=u_{0}\left(y=0,z\right)$ on the wall concentration profile is indicated by the bracket $\left[\phi_{w}\right]$.

Setting z=L and assuming $\phi_{w}\gg \phi_{b}$, it follows in conjunction with the matched asymptotic solution relation $u_{0}\left(L\right)/u^{0}=1-\beta^{0}\langle v_{w}\rangle /v^{*}$ inferred from Equations (44) and (45) that

(A2) $$\langle v_{w}\rangle \frac{R}{D_{0}}\approx \frac{Pe_{R}}{\beta^{0}}{\left[1+c\;\phi_{b}\frac{Pe_{R}^{2}\;{\left(v_{w}\left(L\right)/v^{*}\right)}^{2}}{\phi_{w}\left(L\right)}\right]}^{-1}\equiv \frac{Pe_{R}}{\beta^{0}+\gamma }\;.$$

The function γ introduced on the right-hand-side of the second equality is defined in terms of the left-hand-side. In an ensuing step, we invoke the linear van’t Hoff osmotic pressure expression, $\Pi =(k_{B}T/V_{a})\phi$, for the wall concentration $\phi_{w}\left(L\right)$ at the outlet. This leads to

(A3) $$\frac{\phi_{w}\left(L\right)}{Pe_{R}}\approx \frac{2}{9}\frac{a^{2}}{L_{P}\;R\;\eta }\frac{\Pi (\phi_{w}\left(L\right))}{\langle \Delta_{T}P\rangle }.$$

Substitution of the above expression for $\phi_{w}\left(L\right)/Pe_{R}$ into Equation (A2), and solving for γ, results into

(A4) $$\gamma \approx \beta^{0}\frac{9}{2}\frac{c\phi_{b}L_{p}\eta_{s}\;R}{a^{2}}\times Pe_{R}{\left(\frac{v_{w}\left(L\right)}{v^{*}}\right)}^{2}\frac{\langle \Delta_{T}P\rangle }{\Pi (\phi_{w}\left(L\right))}=\gamma^{0}\;\;g\;,$$

where $\gamma^{0}$ and the function *g* are identified as

(A5) $$\begin{array}{ccc} \gamma^{0} & = & \beta^{0}\frac{9}{2}\frac{c\;\phi_{b}L_{p}\;\eta_{s}\;R}{a^{2}} \end{array}$$

(A6) $$\begin{array}{ccc} g & = & Pe_{R}{\left(\frac{v_{w}\left(L\right)}{v^{*}}\right)}^{2}\times \frac{\langle \Delta_{T}P\rangle }{\Pi (\phi_{w}\left(L\right))}\;. \end{array}$$

In combination with Equation (A2), this leads to Equation (56), with the function *g* given in Equation (A6).
